# Causal deep learning reveals the comparative effectiveness of antihyperglycemic treatments in poorly controlled diabetes

**DOI:** 10.1038/s41467-022-33732-9

**Published:** 2022-11-14

**Authors:** Chinmay Belthangady, Stefanos Giampanis, Ivana Jankovic, Will Stedden, Paula Alves, Stephanie Chong, Charlotte Knott, Beau Norgeot

**Affiliations:** Elevance Health Palo Alto, California, US

**Keywords:** Machine learning, Endocrinology, Type 2 diabetes

## Abstract

Type-2 diabetes is associated with severe health outcomes, the effects of which are responsible for approximately 1/4^th^ of the total healthcare spending in the United States (US). Current treatment guidelines endorse a massive number of potential anti-hyperglycemic treatment options in various combinations. Strategies for optimizing treatment selection are lacking. Real-world data from a nationwide population of over one million high-risk diabetic patients (HbA1c ≥ 9%) in the US is analyzed to evaluate the comparative effectiveness for HbA1c reduction in this population of more than 80 different treatment strategies ranging from monotherapy up to combinations of five concomitant classes of drugs across each of 10 clinical cohorts defined by age, insulin dependence, and a number of other chronic conditions. A causal deep learning approach developed on such data allows for more personalized evaluation of treatment selection. An average confounder-adjusted reduction in HbA1c of 0.69% [−0.75, −0.65] is observed between patients receiving high vs low ranked treatments across cohorts for which the difference was significant. This method can be extended to explore treatment optimization for other chronic conditions.

## Abstract (web summary)

Current treatment guidelines for Type-2 diabetes endorse a massive number of potential anti-hyper-glycemic treatment options in various permutations and combinations. Here, the authors present a causal deep learning approach for more personalized recommendations of treatment selection.

## Introduction

Recent data from the Centers for Disease Control and Prevention estimates that ~13% of the adult population of the United States (US), or about 34 million people, have been diagnosed with diabetes mellitus^1^. When insufficiently managed, diabetes leads to complications including cardiovascular disease, kidney disease, neuropathy, and blindness, any of which can dramatically impair an individual’s quality of life. The high prevalence of diabetes and concomitant complications put a major burden on the US healthcare system in terms of care utilization and costs, with one recent report estimating that one of every four healthcare spending dollars in the US can be directly attributed to diabetes^2^.

Diabetes is typically managed by a combination of lifestyle interventions and pharmacological treatments. For the latter, current guidelines stipulate that unless otherwise contraindicated, initial therapy for type-2 diabetes mellitus (T2DM) should be metformin^3^. If this first-line therapy is insufficient, combination therapy with antihyperglycemic drugs from two or more classes is suggested. There are multiple second-line choices with various risks and benefits, and a clinician may therefore need to attempt multiple treatment combinations before finding one that works for their patient. There have been efforts to determine sequential treatment of diabetes, both with data-driven informatics methods^4^ and with expert-curated guidelines;^5^ however, both of these approaches take into account a few patient-specific characteristics and can be ambiguous in suggesting the next best option for an individual patient. Even when glycemic control is achieved, there is currently no simple way to know whether a different combination might be superior for a given patient, either by providing greater glycemic control, by simultaneously managing comorbidities, or by providing equivalent control at a lower cost, or with fewer total drugs or with fewer side effects. Indeed, the enormous heterogeneity of treatment decisions observed in daily clinical practice is indicative that optimal treatment regimens have not been identified^6^. Given the complexity of diabetes treatment, patients can often benefit from focused subspecialist insights, such as referral to an endocrinologist^7^. However, subspecialist care is resource-intensive and the current shortage of endocrinologists is only projected to grow^8^, thus offering an opportunity for a data-driven understanding of the real-world comparative effectiveness of pharmacological diabetes treatment strategies to help guide T2DM management.

Research on the comparative efficacy and effectiveness of antihyperglycemic drugs has been expanding. The ADOPT trial examined the relative efficacies of three monotherapies using a randomized, double-blind study examining the time to monotherapy failure over multiple years on 4,360 relatively healthy patients between the ages of 30 and 75^9^. Causal analysis methods, such as the frameworks developed by Rosenbaum and Rubin^10^ for observational data are now robust and widespread in clinical research. Meta-analysis^11^ and Network Meta-Analysis^12^ have made it possible to combine results from multiple trials to respectively gain effect insights from a combined pool of patients and leverage both direct and indirect comparisons between treatment arms to reduce measurement uncertainty. These approaches have been applied to a growing body of literature on the effects of T2DM pharmacological interventions, for example to randomized controlled trials (RCTs) and real-world data^4,13^. Although such studies have contributed substantially to clinical knowledge, a comprehensive understanding that reflects the realities of daily practice including diverse patients who may be on more than two classes of antihyperglycemics is still missing.

In recent years, there has been a rapid trend toward digitization in the healthcare industry. Patient medical histories are increasingly recorded in electronic format and claim adjudication systems have become streamlined and more automated. This digitization has led to an explosion in the amount of medical data available to learn from. Concurrently, there have been major advances in the fields of artificial intelligence and machine learning^14^, allowing algorithms to extract complex signals from increasingly larger amounts of data. In medicine, artificial intelligence models have demonstrated human-level performance in interpreting dermatology^15^ and ophthalmology^16^ images. Deep neural networks trained on electronic health records (EHR) have been used to estimate the risk of disease onset^17^, the risk of hospital readmissions^18^, and to forecast the future health state of individuals with complex diseases^19^. It is now possible to use artificial intelligence to extract meaningful insights from large-scale observational studies, which can be extended to potentially infer causal relationships.

Here, an approach is demonstrated that combines deep learning, causal inference, and network meta-analysis^12^ (NMA) to estimate the real-word comparative effectiveness of combination therapies for T2DM in clinically stratified high-risk sub-populations. Using the change in levels of glycated hemoglobin (HbA1c) as the primary outcome of interest, effectiveness was measured by estimating confounder-adjusted average treatment effects (ATE) of each treatment strategy relative to other treatments, at the level of drug classes, observed using a nationwide cohort of patients with poorly controlled T2DM. This work departs from previous research in several important ways: (i) it is, to our knowledge, the first study to extend beyond single or dual therapies and compares all treatment regimens observed in the data without imposing restrictions on the number of drug classes; results on combinations of up to five drug classes are reported here; (ii) the analysis was performed on 10 cohorts stratified based on clinical variables to make the rankings more personalized; (iii) a recently developed deep-learning-based propensity score model was used for causal analysis that scales well to large multi-arm observational studies; and (iv) a sensitivity analysis was performed on held-out test data in order to assess the extent to which the comparative effectiveness rankings were meaningful and broadly generalizable. With further development and prospective validation, these rankings for combination therapies could form the basis of a tool to complement/enhance guideline-based practice and help clinicians make personalized data-driven decisions when deciding the next step in treatment for their high-risk patients.

## Results

### Inclusion criteria

**Fig. 1:**
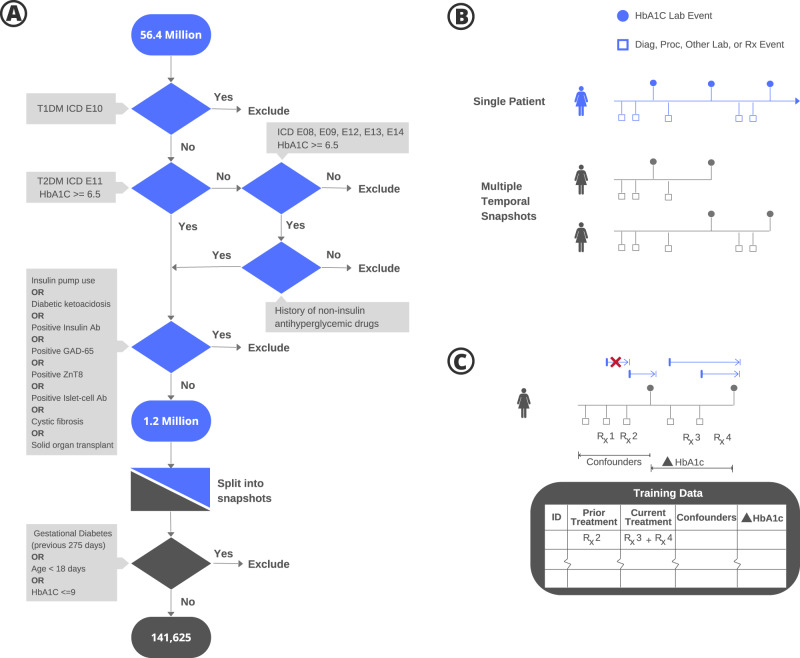
Study cohort definition and data preparation. **a** Clinical filters were designed to identify patients with T2DM (1.2 million individuals) and retain only those with well-established, high-risk disease. **b** Each patient’s health history was split into a series of temporal snapshots, determined by an HbA1c ≥9% lab measurement and ending at the subsequent HbA1c for each patient. A snapshot consisted of a pair of HbA1c lab events. The first of these is referred to as the index HbA1c lab event and the period between the two lab events is the observation period. Only snapshots where the duration between the lab pairs was between 90 and 365 days were retained and the rest were excluded, resulting in a final study population of 141,625 patient snapshots. All further analyses were conducted at the level of patient snapshots. **c** A patient was considered to have been treated by a particular antihyperglycemic drug at the time of a given HbA1c lab event if it was prescribed prior to the lab and if the number of days’ supply (blue arrows) extended past the lab date. When multiple such drugs existed, the individual was considered treated by the combination of these drugs. Prior treatment was the regimen used to treat the individual in the period prior to the observation window between the two labs.

The data for this study came from health insurance claims of 56.4 million individuals collected over a 5-year period (see Methods). The claims contain records of diagnoses made during doctor visits, procedures performed in in-patient or out-patient medical centers, lab tests ordered and their results, and drug prescriptions including dosage and refill information. The set of claims for an individual, therefore, serves as a succinct historical record of that individual’s state of health. Clinical filters (Fig. 1a) were used to identify a subpopulation of 1.2 million patients with T2DM. Temporal “snapshots” (Fig. 1b) of patient health histories, beginning with a given HbA1c event and ending with the next subsequent HbA1c lab measurement for each pair of HbA1c events in a patient’s timeline, were generated for each person in this subpopulation from their available history of medications, diagnoses, procedures, and relevant laboratory values to assess the treatment strategies and resulting causal effect calculations (Fig. 1). The combinations of medications, at the level of drug class, that a patient was filling during the time of interest was considered a treatment. We evaluated changes in HbA1c regardless of whether the treatment regimen changed during the snapshot. If a regimen did change during a snapshot, the change in A1c was attributed to the new regimen. Snapshots were filtered for those with an initial HbA1c ≥9% to target high-risk patients who were clearly above goal^20^ and could most benefit from treatment insights. Causal inference analyses were conducted on a final study population of 141,625 patient snapshots.

### Clinical cohort definition

Because target HbA1c varies by a patient’s age and health status^20^, patient snapshots were assigned to 1 of 10 clinical cohorts on the basis of each patient’s age and a number of additional chronic health conditions at the time of the HbA1c index event, as well as by insulin status at the time of the index HbA1c. Cohorts by age/comorbidities were chosen to prevent the algorithm from inappropriately optimizing low HbA1c in groups for which sub-target HbA1c may be due to dangerous hypoglycemia, and cohorts by insulin status at the time of index HbA1c were chosen as a proxy for hypoinsulinemia, which would be dangerous to misclassify but is not otherwise well-captured in the available data. The number of snapshots present as well as the number of treatment strategies that cohorts were exposed to tended to decrease with age and disease burden (Table 1) though these trends did not decrease monotonically. There were 81 unique treatment regimens identified across all clinical cohorts. The number of distinct treatment strategies observed in a cohort was correlated to the number of patient snapshots present; the larger the population, the more unique treatment strategies were observed.

**Table 1:** Definitions and characteristics of T2DM patient cohorts

Cohort	Insulin status	Age	CCI	Number of snapshots	Number of patients	Number of treatments
A	Non-user	<65 years	≤2	54415	42676	69
B	Non-user	<65 years	>2 & <5	21342	16570	50
C	Non-user	<65 years	≥5	9164	7121	37
D	Non-user	≥65 years	<5	8971	7163	30
E	Non-user	≥65 years	≥5	4339	3504	18
F	User	<65 years	≤2	13422	7025	43
G	User	<65 years	>2 & <5	13057	9356	43
H	User	<65 years	≥5	8973	5878	35
I	User	≥65 years	<5	3661	2681	15
J	User	≥65 years	≥5	4281	3018	19

CCI (unweighted) Charleson Comorbidity Index; Snapshots divided into cohorts by the patient’s history at the time of the index HbA1c laboratory event as defined in Fig. 1b. Number of treatments refers to the number of unique treatment strategies (including combinations of drugs) observed in the cohort as detailed in Fig. 1c. Also shown is the number of patients in each cohort. Bolding of cohort names and headers for clarity.

### Characteristics of the study population

Table S1 summarizes variable values for the study population at the snapshot level.

The snapshot population had a mean age of 55 years, with baseline HbA1c, estimated glomerular filtration rate (EGFR), and creatinine lab values of 10.5%, 94 mL/min/1.72m^2^, and 0.9 mg/dL respectively. Data on race and ethnicity was available for only 28% and 16% of the patients, respectively, and no income data was available. Since these variables can act as confounders, information on the racial makeup and income levels in the patient’s zip code tabulation area (ZCTA) were used as proxies. This demographic data came from the 2017 American Community Survey published by the US Census Bureau^21^. Neighborhood median incomes ranged significantly with a median value of fifty-five thousand dollars annually. White, Black, and Asian populations were well represented with Whites being in the majority. As expected, within a population of patients with significantly elevated blood sugar, a wide range of comorbid conditions were present, with obesity, heart disease, COPD, and renal disease being most prevalent. For model training and validation purposes, patient snapshots were divided into training (80%) and test (20%) sets that matched statistically on all variables.

### Causal modeling of optimized treatment ranking by subpopulation

**Fig. 2:**
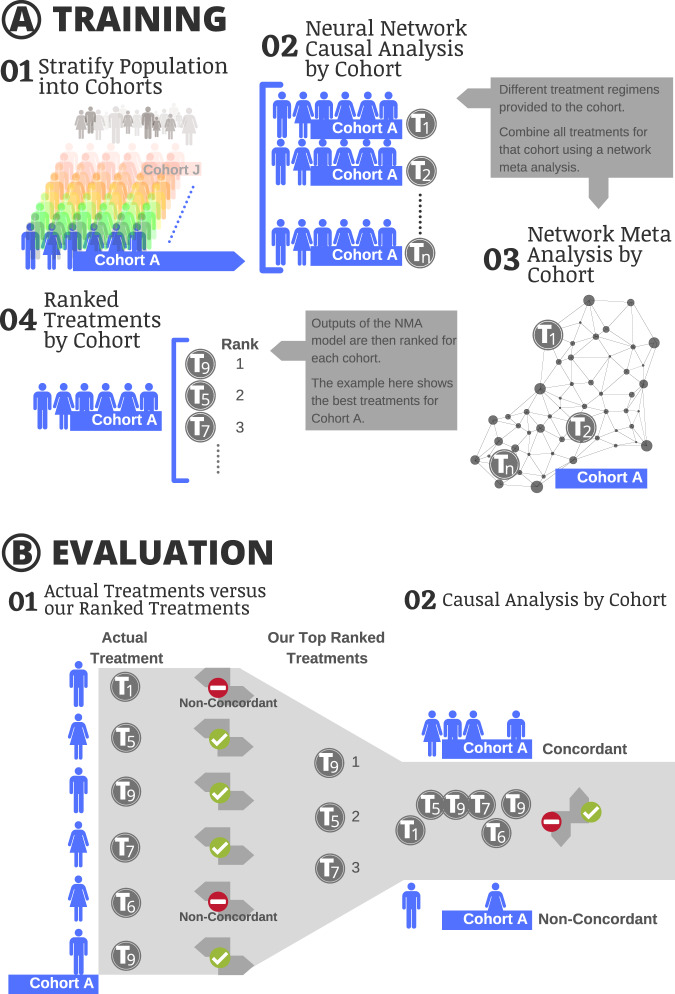
Schematic of ranking generation and analysis. Snapshots were split into training (80%) and test (20%) datasets. Snapshots were stratified into 10 clinical cohorts based on age, number of comorbidities, and prior insulin use (Supplementary Table 1). For each clinical cohort, all treatments with cohort size >35 were selected and case-comparator observational studies were performed comparing every treatment with every other treatment using BCAUS, a neural-network-based propensity score model for causal inference. A densely connected network graph was constructed with treatments as nodes and edges connecting treatments via measured Average Treatment Effect (ATE) values. Bayesian Network Meta-Analysis (NMA) was performed to compute network-synthesized ATEs compared against a baseline treatment which was set to Metformin (the first-line therapy for T2DM). Treatments were sorted by their Surface Under the Cumulative RAnking curve (SUCRA) scores^42^ to generate a ranked list of treatment strategies for the cohort. To gauge the effectiveness of the ranking procedure, each cohort in the test set was divided into a concordant group consisting of patient snapshots where the prescribed treatment was one of the top-three ranked treatments and a non-concordant group where the prescribed treatment did not match any of the top-three ranked treatments. The difference in HbA1c between the concordant group and the non-concordant group was used to estimate the confounder-adjusted ATE of the comparative effectiveness treatment rankings.

A schematic of the modeling approach to generate treatment rankings is shown in Fig. 2 and described in Methods. Significant differences existed in the underlying covariate distributions between treatment and comparator arms in the observational data but were successfully balanced through the BCAUS^22^ methodology (Supplementary Fig. 1). The confounder-adjusted causal relative effect (see Supplement) of each treatment strategy compared to other treatments was calculated independently for each cohort (Fig. 2). Network Meta-Analysis was performed and treatment strategies were ranked based on network-synthesized causal reduction in HbA1c.

The top-10 most effective treatment strategies for each cohort (Supplementary Tables 4 and 5), revealed that the highest-ranked treatment strategy was unique to each cohort (see Supplementary Fig. 14). Note that a change in treatments that occurs between the two HbA1c lab events is attributed to the new regimen (at the time of the terminal HbA1c) and not to the prior one (from the time of the index HbA1c), which is why non-insulin regimens may appear in the insulin-using groups (which are defined by insulin status at the time of index HbA1c). Also note that these rankings are by point estimates, which may have overlapping uncertainty intervals (Supplementary Figs. 2–11). GLP-1s and metformin, both known to be highly efficacious for blood glucose control^23^, are the only classes to appear as monotherapies in any group’s top ten ranked treatments, though they only appear for half of the cohorts and never higher than position five. A complete listing of the rankings for all treatment strategies across all cohorts can be found in the Supplement (Supplementary Fig. 12) as well as the measured causal effects, confidence intervals, and sample sizes for all treatment strategies in each cohort (Supplementary Figs. 2–21).

### Causal effect of treatment rankings on HbA1c reduction

**Fig. 3:**
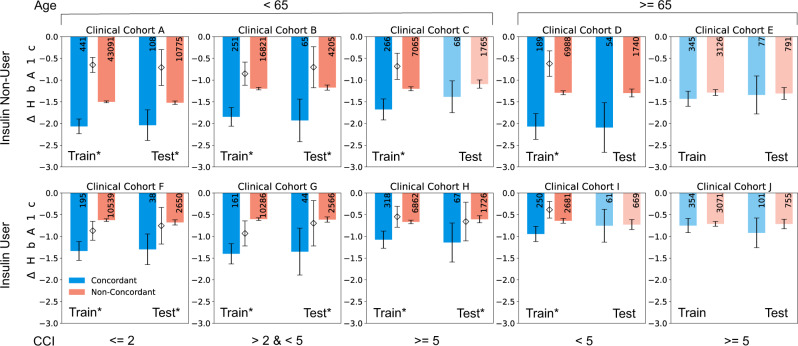
Causal effect of treatment rankings on HbA1c reduction. Evaluations for concordant (blue) and non-concordant (red) groups for all clinical cohorts. An individual is considered concordant if their current treatment matches one of the top-three recommendations for their clinical cohort and non-concordant otherwise. Training and test set results are shown. The * denotes that the confounder-adjusted Average Treatment Effect (ATE) of the comparative effectiveness treatment rankings between concordant and non-concordant groups is statistically significant (*p* < 0.05) by independent two-sample *t* test (see Ranking Validation Procedure section of the Methods). Diamonds show ATE values for cases that are statistically significant (asterisk). Error bars represent 95% confidence intervals. Numbers on the bars denote the number of patient snapshots in each group. CCI (unweighted) Charlson Comorbidity Index.

Significant differences in patient outcomes were observed between the top-three treatments (representing 2.4% of all snapshots) and all other choices (Fig. 3), with an average confounder-adjusted reduction in HbA1c across cohorts of 0.69%. The differences were significant clinically as well as statistically, persisted even after controlling for differences between patients that received highly ranked choices versus others, and generalized extraordinarily well to the test cohorts. A sensitivity analysis revealed a consistent relationship between top three, ranks 4–10, and 11 and below treatment strategies (Supplementary Fig. 13).

### Ranking group prescription patterns in real-world observational data

The distribution of high-ranked treatment strategies provided to patients in each clinical cohort in the study population of patients with poorly controlled diabetes (Supplementary Table 2) was evaluated. Across all cohorts, the average treatment rank per snapshot was 28. The lowest rates of concordance were observed among the younger or relatively healthier cohorts. In 62% of cases where a single patient had multiple associated snapshots, differences in treatment strategies were analyzed between consecutive snapshots. The overall incidence of these patients switching treatments between snapshots was 35 percent. When patients switched treatments, 51 percent of those switches led to a new treatment with a better rank (with an average improvement of 13 positions of rank), while 49 percent of switches led to treatments with a worse rank for the patient (with an average decrease in rank of 12). The mean change in treatment rank across all changes was an improvement of 1 position.

## Discussion

In this study, antihyperglycemic treatment strategies for patients with an HbA1c ≥9% were examined over a five-year period in a nationwide cohort of US patients with T2DM. Over 80 different strategies of drug class combination were observed, ranging from monotherapy to combinations of five distinct drug classes. This enormous heterogeneity persisted even after accounting for age, a number of comorbidities, and status of insulin dependence. A network meta-analysis using deep causal models was performed on the cohort’s observational data to rank treatment strategies for ten clinical cohorts based on effectiveness in lowering HbA1C in the high-risk population. The rankings differed between each of the cohorts and they generalized well to snapshots in the held-out test set. Top-three ranked treatments were clinically and statistically better at lowering HbA1c than other choices for most cohorts. There were considerable differences between which treatments were best for each of the clinical cohorts (Supplementary Fig. 14), though the specific class and combination were cohort dependent. Therapeutic classes known to provide secondary cardioprotective benefits, such as SGLT2’s and GLP-1’s, feature prominently in the top ten choices for each cohort. Although cardioprotection was not evaluated in this study, this finding may indicate that there is no need for a trade-off between glucose control and cardioprotection. Additionally, although no monotherapy was the top-ranked treatment for any cohort, the rankings clearly show that simply adding more drug classes^24^ to a patient’s regimen is not uniformly best for HbA1c reduction, consistent with prior literature showing decreasing adherence and worse outcomes with the increasing complexity of medication regimens^25,26^, as well as the lack of association between polypharmacy (>4 drugs) and improved A1c control^27,28^ in patients with diabetes. In our data, treatment switches, when they occurred, moved patients into lower-ranked strategies as often as they resulted in higher-ranked strategies. Interestingly, in the insulin non-user groups, insulin-containing regimens tend to rank poorly, suggesting poor real-world effectiveness^29^ of a medication known to have high efficacy in Randomized Controlled Trials^20^.

That treatment strategies for T2DM are massively heterogeneous is well known. While Hripcsak et al.^6^ observed that 10% of diabetic patients in their international study had treatment pathways that were unique specifically to that individual, the authors hazard that the variability was not a sign of personalization but rather that “it may point to a failure of the field to converge on an effective treatment”. To our knowledge, this is the first study to examine comparative effectiveness between all observed treatment strategies in multiple clinically-relevant real-world cohorts. The monotherapy^9^ and dual-therapy^30^ results found in this work are reasonably consistent with prior published results, which were limited to those two options. However, differences in cohort sizes and inclusion criteria make direct comparisons difficult. For example, the ADOPT trial^9^ contained fewer than 5,000 participants and excluded patients with more advanced diseases that would not be eligible for monotherapy. Mearns et al.^30^. combined all patients from dual-therapy trials, regardless of age, disease severity, or comorbid conditions, making it impossible to directly compare results to the clinically stratified cohorts examined here. Rosenstock et al.^31^. evaluated the effectiveness of linagliptin vs glimepiride as second or third-line therapy in achieving goal HbA1c as a secondary outcome of a randomized trial, but the other medications in the combination were not evaluated.

Although no treatments were censored, no treatment strategies were found to be in violation of current standard-of-care guidelines^32^. While it is reassuring that guidelines are generally followed, it also reinforces the concern that guidelines may be insufficient at guiding treatment choices for blood sugar reduction. Instead of contradicting current best practices, the findings provide clarity on which strategies may be best when the guidelines allow many to choose from. It is perhaps not surprising that few patients were on a treatment that may be most effective for them. Patients with highly elevated HbA1c are, by definition, those who have not yet found a treatment strategy that works within their circumstances to control their blood sugar. Additionally, since the current guidelines unilaterally suggest a progressive approach from mono to dual therapy, followed by experimentation within dual-therapy before adding more drug classes, there is a diffusion effect that necessitates a long time until sufficient experimentation has occurred to identify a good strategy for many patients.

Importantly, the results of network meta analyses (NMA) must be interpreted with care. Mbaugbaw et al.^33^. specifically review common pitfalls with the clinical interpretation of NMA results, especially SUCRA rankings. For example, they note the tendency to interpret a ranked list of treatments as a definitive hierarchy of best options, without contextualizing the quality of the underlying data, the inherent uncertainty surrounding each rank position, or that the NMA evaluates treatments only based on one component of an often multifaceted treatment decision. In our study, our major clinical finding is that the average treatment effect of a top-three treatment choice is significantly greater than a lower-ranked treatment choice in multiple cohorts. The absolute rankings by point estimate are provided for examination in Supplementary Table 4, with contextual uncertainty metrics for each treatment by cohort additionally provided in the supplement. As noted by Mgbaugbaw et al.^33^, our NMA is limited to one component of treatment decisions- the potential change in A1c- and does not evaluate other aspects of clinical treatment decisions such as side effects, secondary benefits, or patient preferences; the rankings provided here should not be interpreted as definitive treatment recommendations.

Although this work has potentially significant clinical value for developing a clinical decision support tool, and may even provide the signal necessary for the field to identify effective treatments that Hripcsak et al. have called for^8^, there are several important limitations. As with any observational trial, unobserved confounding may affect results and so the results will need to be validated prospectively. Also, the inverse probability weighting method creates pseudo-populations in case and comparator arms that are balanced in observed confounders. While this method decouples case-comparator observational studies that (prior to weight adjustment) may share a common cohort, it is possible that residual correlations exist and the variances in the network-synthesized ATEs are underestimated. Additionally, effectiveness was defined exclusively on the grounds of HbA1c reduction. This choice is reasonable given that HbA1c as a surrogate endpoint is the most used outcome for clinical trials and that our study population is comprised of patients with highly elevated blood sugar, however, there are additional clinical endpoints, particularly those related to cardiovascular outcomes, that are relevant for patients with diabetes. For example, the low concordance with a top-ranked treatment could potentially be due to physicians prioritizing cardioprotective or nephroprotective medications at the expense of glucose-lowering medications. However, the strong presence of treatments with secondary protective effects (i.e., SGLT2 inhibitors, GLP-1 agonists) in the top-ranked choices may indicate that while the effects on cardiovascular and renal outcomes are not captured here, the treatment choices that are most effective for glycemic control are top choices for secondary protective effects as well. Nonetheless, conclusions about how treatment protocols that utilized the rankings derived here would impact these endpoints cannot be drawn from this work. A second limitation is that impact on HbA1c was only calculated at the follow-up measurement after treatment was assigned (a 6-month median window). This time period is sufficient to see the effects of medication changes considering the half-life of hemoglobin, including the slower-acting thiazolidinediones^20^, but may not be perfectly indicative of long-term trends and tolerability. We also do not consider treatments prior to the time period surrounding a given snapshot. Given the length of time it takes for diabetes-related complications to develop, causal attribution to specific treatment strategies is clouded by the many patient-related factors that can change over such a length of time, such as the course of treatment. However, a longer study period that tracks clinical endpoints as well as laboratory endpoints is desirable and could be feasible as datasets such as this grow over time. Future studies could leverage our methodology to define effectiveness by distance from a specified HbA1c value for each clinical subpopulation, instead of absolute HbA1c reduction. Alternatively, maximal risk reduction for microvascular or macrovascular outcomes could be used as the endpoint instead of HbA1c. However, such investigations would be most robustly served by a prospective study tracking the impact on multiple clinical endpoints from prescribing high-ranked treatment strategies to achieving cohort-specific targets.

We envision that this work can be the basis for the development of a clinical decision-support tool for choosing or augmenting diabetes treatments in patients with T2DM. Although the treatment rankings presented in this work are fixed to the clinical population defined above, the method used to identify top treatment regimens could be applied in validated populations to supplement guidelines to support many different approaches to decision-making. For example, based on patient needs and clinician preferences, some may choose not to prescribe the highest-ranked treatment but instead the highest-ranked option that involves the smallest change from the current regimen. Alternatively, for patients for whom compliance may be a concern, selecting a treatment that optimizes the rank with the fewest number of total drugs would be an option. Findings could also be filtered to avoid contraindications (e.g., based on most recent EGFR, hypoglycemia risk, or patient allergies), minimize costs, or avoid injections. For every highly ranked strategy in our study that contained many different drug classes, there was usually a simpler combination with nearby rank. Additionally, clinicians likely have access to information that the model does not, such as BMI or duration of diabetes, and thus a non-prescriptive, filterable decision support tool will allow them to overcome this limitation. The enormous variety of ways in which these comparative effectiveness rankings could be utilized may be best leveraged by software with a performant, intuitive user interface to return the optimized results for a given patient target. Such software could also provide additional metrics captured in this study, like the number of patients observed on each treatment strategy and the clinical and demographic parameters associated with each person, which are not possible to display in the context of individual patients within a manuscript like this. Additional convenience functions, such as the removal of contraindicated treatments from the rankings list for each individual patient may be desired. As with any clinical decision support tool, evaluation and optimization will be an ongoing process to ensure no undesirable effects^34^.

Taken together, these findings have important implications for personalizing care for chronic health conditions. The approach outlined here represents a concrete step towards a functional learning healthcare system, and it is immediately extensible to other conditions beyond diabetes mellitus that have complex pharmacological treatment patterns such as hypertension, asthma, chronic obstructive pulmonary disease, depression, and congestive heart failure. By forestalling adverse events that arise from unmanaged chronic diseases, such learning systems could greatly reduce patient suffering and lead to significant reductions in healthcare costs.

## Methods

### Study cohort definition and data preparation

This manuscript reports findings that were obtained as a part of healthcare operations quality improvement using only aggregated results of the analysis; no individually identifiable information (protected health information or otherwise) was used in the development of the manuscript and the work was deemed not human subjects research by the Anthem Office of General Counsel (OGC). Electronic health records were analyzed for 56.4 million members from a healthcare plan population between 1 December 2014 and 1 January 2020 to determine the average treatment effect of diabetes medications on HbA1c. The records included approximately five billion insurance claims (for diagnoses, procedures, and drug prescriptions or refills) as well as lab test results for the associated patients. Not all patient records spanned the entire five years. Clinical filters were designed to distinguish between major sub-types of diabetes, and patients with Type I diabetes, anyone under 18 years of age, or gestational diabetes were excluded from the study (Fig. 1a). Individuals with histories of diabetes ketoacidosis, cystic fibrosis, or solid-organ transplants were also excluded as a safety precaution because they are highly complex patients who would clearly benefit from subspecialist care and the rankings developed herein are targeted towards PCPs managing typical patients with T2DM. Snapshots with HbA1c’s below 9% at the initial HbA1c were also removed to focus on high-risk patients who were clearly eligible for treatment strategies beyond first-line based on the Center for Medicare and Medicaid Services definition^35^ of poor glycemic control as well as because an HbA1c > 9% is clearly above goal for almost all patients^20^. This filtering resulted in a study population of 104,992 unique individuals.

The health status of any individual evolves with time. Since the study period in our work spanned several years, to properly account for this evolution, each individual’s health history was split into a series of temporal snapshots as shown in Fig. 1b. Each snapshot was determined by an index HbA1c ≥9% lab measurement and terminated at the subsequent HbA1c for each patient, with a lookback period to the patient’s first healthcare event on record. The time period between the two labs was considered the observation period. The age of the individual in a particular snapshot and any clinical covariates that were treated as confounders were measured as of the date of the first lab of the pair. Individuals with only a single HbA1c lab report were excluded. Only snapshots where the observation period was between 90 and 365 days were retained, and the rest were excluded, resulting in a final study population of 141,625 patient snapshots, with each patient contributing on average 1.3 snapshots for analysis. Snapshot duration was not otherwise considered in the model. As shown in Fig. 1c, an individual was considered as treated by a particular antihyperglycemic drug at the time of an HbA1c lab event if it was prescribed prior to that lab and if the number of days of supply plus a grace period of 30 days (for non-adherence) extended past the lab date. If a regimen changed during a snapshot, the change in HbA1c was attributed to the new regimen. Because HbA1c reflects glycemic control over a period of approximately 90 days^36^ and the half-lives of anti glycemic medications are on the order of hours to days^37^, only current treatments were considered in the model. Given that metformin is the consensus first-line therapy and as our goal was to compare efficacy between treatment regimens, patients on no treatment were excluded. Medications taken prior to the pre-snapshot period were not included in the model. When multiple drugs existed within the inclusive dates, treatment was considered the combination of these drugs. Diabetes drugs were identified only by their class names (e.g., SGLT2 inhibitors, sulfonylureas, etc.) and non-diabetes drugs were excluded. All further analysis was performed on the pseudo-population of patient snapshots.

Many clinical and social factors are known to be associated with diabetic treatment selection and HbA1c outcomes. For example, kidney function as well as the presence of various comorbid conditions may result in contraindications for certain antihyperglycemic drug classes and may also influence the HbA1c value that the prescribing clinician targets for an individual. Additionally, social determinants of health (SDoH) such as patient race, income, and location are known to influence both treatment selection and health outcomes. In order to control for these confounding factors so that an accurate estimate of the causal effect of treatment strategies could be obtained, all comorbidities present in the history of each patient were included using diagnostic definitions defined by the Charlson Comorbidity Index (CCI)^38^, as well as the most recent EGFR and creatinine values at the time of each snapshot. Race is known to be reported at very low levels both within EHRs and claims data. Accordingly, census-derived data on the racial and economic profiles of each patient’s neighborhood using zip codes was used. These are weak surrogates for true SDoH markers, but we believe that including them is still significantly better than ignoring SDoH completely from large-scale clinical studies. Missing data were imputed to the mean (Supplementary Table 3); no age or sex data were missing. Supplementary Table 1 provides the summary statistics of all covariates that were treated as confounders for causal inference.

#### Causal inference modeling

Several methods for the causal inference analysis were considered for use. Because there are multiple possible combinations of treatments, the number of head-to-head comparisons that need to be performed is extremely large. Propensity score matching^11^ or weighting^39^ methods are widely used for observational studies but are considered “do-it-yourself,”^40^ in that the propensity score model must be checked for correct specification after it is trained, and, when incorrectly specified, it has to be retrained by modifying model parametrization or feature engineering. Automated methods like Bayesian Additive Regression Trees^41^ have yielded good performance on benchmark datasets^40^, but rely on Monte Carlo sampling and are therefore prohibitively slow for the number of comparisons necessary in this study. Recently, a technique was introduced^22^ called BCAUS (Balancing Covariates Automatically Using Supervision) that scales well to massive multi-arm studies. BCAUS consists of a neural-network propensity model that is trained using a joint loss given by

$${L}_{{TOTAL}}=\,{L}_{{BCE}}+\nu \mu {L}_{{BIAS}}.$$

The first term, ${L}_{{BCE}}$, is a binary cross-entropy loss which penalizes incorrect treatment prediction, while the second, ${L}_{{BIAS}}$, is a loss term which explicitly tries to minimize imbalance between inverse probability weighted covariates. Details of the training process are described in Supplementary Materials and a comparison with other state-of-the-art neural-network-based methods on benchmark datasets has been described elsewhere^22^. For each pairwise comparison between diabetes treatments, a separate BCAUS model was trained. The propensity score outputs of trained models were used to estimate average treatment effects using Inverse Probability of Treatment Weighting (IPTW). A bootstrapping procedure was used to compute standard errors and confidence intervals (see Supplement). The input data for NMA consisted of the estimated ATEs and standard errors.

#### Network meta-analysis

An ATE value measured via a direct causal comparison between two treatments has to be consistent with values that are indirectly estimated by comparing each treatment of the pair with intermediary treatments and then computing differences. Separate network graphs were constructed for the 10 clinical cohorts where every treatment node was connected with every other treatment node. Edges representing observational studies where all confounding covariates were not balanced were trimmed and Bayesian NMA was performed over the resultant graph. We used a random-effects model, set uninformative priors, and used a Markov Chain Monte Carlo (MCMC) sampling procedure to construct posterior distributions of ATE values for all treatment pairs. To determine relative ranks, samples were drawn from the posterior predictive distributions of ATE values of all treatments compared against metformin, which was set as the baseline treatment. For each draw, treatments were ranked in ascending order of ATE values (i.e., higher ranks for more negative values), and a mean rank was computed for each treatment across all draws. This mean rank was normalized to compute the Surface Under the Cumulative RAnking curve (SUCRA) score^42^. Treatments were ranked in descending order of SUCRA scores such that the treatment that reduced HbA1c by the largest amount relative to metformin had the highest rank. This ranked list of treatments applies to all members of a given cohort; within-cohort treatment heterogeneity is not further accounted for by the model. Further details of the training procedure are available in Supplementary Materials.

#### Ranking validation procedure

To investigate the degree to which the rankings generalized to new patients while generating an estimate of the improvement to HbA1c over existing practices if rankings were used to guide treatment decisions, outcomes between patients whose physicians happened to have prescribed a top-3 ranked treatment choice for them versus selecting any other treatment option were compared retrospectively. Snapshots in each clinical cohort were divided into concordant cohorts (where the actual prescribed treatment matched one of the top-3 recommendations) and non-concordant cohorts (where a patient was provided any treatment ranked four or lower). Differences in the mean change in HbA1c between the concordant and non-concordant groups were calculated for all cohorts for both training and test datasets. If the difference in means was found to be statistically significant (using an independent two-sample *t* test), an additional confounder-adjusted case-comparator study was performed between the cohorts to measure whether the differences in means were directly attributable to the differences in treatment strategy ranks.

To further investigate if the rankings demonstrate an internally consistent effect, a sensitivity analysis was performed by splitting patient snapshots of each cohort in the training dataset into three concordance cohorts: (i) the “top” cohort is concordant with treatments ranked 1–3; (ii) the “middle” cohort is concordant with treatments ranked 4–10, and (iii) the “bottom” cohort is concordant with treatments ranked 11 and below. Confounder-adjusted ATE values were estimated, comparing the top versus bottom groups and the middle versus bottom groups. If the ranks are internally consistent, an effect where-in the top outperforms the middle and the middle outperforms the bottom would be expected. The ranks were internally consistent as shown in Supplementary Fig. 13.

### Reporting summary

Further information on research design is available in the Nature Research Reporting Summary linked to this article.

## Supplementary information

Supplementary Information

Reporting Summary

## Data Availability

The raw data are protected and are not available due to data privacy laws and commercial interests. Investigators with an academic affiliation may contact the corresponding author for data access for the purposes of validating the above findings. Requests will be processed within 60 days.

## Code availability

The code for the method used in this study is freely available at https://github.com/gstef80/bcaus_nma.
