# Supplementary material for: Causal deep learning reveals the comparative effectiveness of antihyperglycemic treatments in poorly controlled diabetes
Source: Nat Commun. 2022 Nov 14;13:6921. doi: 10.1038/s41467-022-33732-9 (PMC9663714; doi:10.1038/s41467-022-33732-9)
Supplement: Supplementary file 1 — Supplementary Information [file 41467_2022_33732_MOESM1_ESM.pdf]

Supplementary Materials

Causal Analysis with BCAUS

As described in the main text, the BCAUS[1] model is a neural network-based propensity-score model that is trained using a joint loss that consists of two components: $L_{Total} = L_{BCE} + \nu\mu L_{BIAS}$. The first loss term helps the model predict treatment assignment (as in conventional propensity score modelling) while the second term depends explicitly on the imbalance between intervention arms in the data, forcing the network to learn parameters that minimize this imbalance. Here μ is the scalar ratio of L_{BCE} to L_{BIAS} that is detached from the computation graph i.e. gradients are not evaluated for μ with respect to the network parameters during backpropagation. The relative contribution of each loss component is tuned using hyperparameter, ν. The cross-entropy loss is calculated as:

$$L_{BCE} = \sum_i t^{(i)} \log\left(p^{(i)}\right) + \left(1 - t^{(i)}\right) \log\left(1 - p^{(i)}\right) \qquad (1)$$

Here $t^{(i)} \in \{0,1\}$ is the treatment given to individual i. To compute the bias loss, the propensity score $p^{(i)}$ is used to compute the inverse probability weight (IPW):

$$w^{(i)} = \frac{t^{(i)}}{p^{(i)}} + \frac{1 - t^{(i)}}{1 - p^{(i)}} \qquad (2)$$

The mean squared error of the M covariates weighted according to Eq. S2 is used to calculate the bias loss:

$$L_{BIAS} = \frac{1}{M} \sum_{j=1}^{M} \left(\frac{\sum_i t^{(i)} w^{(i)} x_j^{(i)}}{\sum_i t^{(i)} w^{(i)}} - \frac{\sum_i (1 - t^{(i)}) w^{(i)} x_j^{(i)}}{\sum_i (1 - t^{(i)}) w^{(i)}} \right)^2 \qquad (3)$$

The two terms in the equation above represent the weighted means of the covariates for the treatment and control groups respectively. To assess balance, the standardized mean difference Δ_j for each covariate j is computed according to:

$$\Delta_j = \frac{\left|\underline{x}_{j,\text{treatment}} - \underline{x}_{j,\text{control}}\right|}{\sqrt{\frac{\left(s_{j,\text{treatment}}^2 + s_{j,\text{control}}^2\right)}{2}}} \qquad (4)$$

Here $\underline{x}_j$ is the weighted mean of x_j and s_j^2 is its weighted variance with weights assigned according to Eq. 3. The standardized mean difference can also be defined for the raw data without the weights, in which case x_j and s_j^2 represent the unweighted mean and variance respectively. Here (4) is not used in the model training but is used as a diagnostic to check if covariates have been correctly balanced. Further details of the BCAUS model and comparison on benchmark datasets have been reported in Belthangady et al.[1] The BCAUS model is implemented in Python using the PyTorch neural networks library. Each BCAUS model consists of two hidden layers with the number of neurons in each layer set to twice the number of input covariates. Rectified Linear Units (ReLU) activation is used for all layers except the last layer consisting of single neuron which uses sigmoid activation. In addition to the two loss terms, an L2 penalty is imposed on the network parameters for regularization. To further reduce overfitting, dropout regularization is used in the neural network. Code for BCAUS definition and training has been published elsewhere. We performed cross-validation using a grid of parameters for nu (values: 1, 2, 3, 4) and dropout (0, 0.15) to decrease the risk of overfitting. The learning rate (i.e., step size at each iteration) was set to 0.001, and the networks were trained for 1000 epochs (i.e., training iterations). An early-stopping procedure was implemented where training terminated if all covariates remained balanced (i.e. standardized mean difference < 0.1) for more than 10 epochs.

For each clinical subgroup, all treatments with more than 35 treated individuals were chosen and BCAUS models were trained comparing every treatment with every other treatment. For a treatment pair i and j the estimated ATE values should be antisymmetric i.e. $ATE_{ij} = -ATE_{ji}$ and for n treatments, $n(n-1)/2$ pairwise comparisons should suffice. However, since the propensity scores output by BCAUS are not calibrated probabilities, a small deviation from this symmetric property (with differences much smaller than the standard error) is observed in practice. Therefore ATE_{ij} and ATE_{ji} were computed separately and a total of $n(n-1)$ BCAUS models were trained. Prior to training all continuous covariates in each clinical subgroup were Z-scored to have zero mean and unit standard deviation. Inverse probability weights were computed according to the weight stabilization method described in Reference

S2 and ATE values were computed. A bootstrapping procedure for IPTWs was used to estimate the standard error for the ATE values. Inverse propensity weighted outcomes were picked at random and with replacement from the dataset and ATEs were computed between control and treatment individuals in each draw. The standard deviation of ATE values across 100 draws was reported as the standard error. ATE values and their standard errors for all pairwise treatment combinations were computed for each clinical subgroup and NMA was performed using the procedure described below. Only case-comparator studies where all covariates were balanced were included in the NMA.

Network Meta-Analysis

Network Meta-Analysis was performed with Python code developed using the PyMC3 probabilistic programming library. The network graph was encoded as a random-effects model:

$$\mathrm{ATE}_{ij} \sim \mathrm{Normal}\left(\delta_{ij}, \mathrm{se}_{ij}^2\right) \quad (5)$$

$$\delta_{ij} = d_{ij} + \tau\ \mathrm{Normal}(0,1) \quad (6)$$

$$d_{ij} = d_i - d_j \quad (7)$$

$$\tau \sim \mathrm{HalfCauchy}(5) \quad (8)$$

$$d_i \sim \mathrm{Normal}(0, 15 * (|\mathrm{ATE}|)\ ) \quad (9)$$

Here ATE_{ij} is the ATE value measured by comparing treatment i against treatment j and se_{ij} is the corresponding standard error, d_i is the ATE of treatment i relative to the baseline treatment with $d_{\mathrm{baseline}} = 0$. Uninformative priors were set for τ (the standard deviation) and d_i with the standard deviation for the sampling distribution of the latter set to 15 times the maximum absolute value of measured ATEs. A non-centered parameterization was chosen for the model because Markov Chain Monte Carlo (MCMC) samplers have difficulties sampling from the “Neal’s funnel” that can lead to divergent trajectories and biased results. A No U-Turns Sampler (NUTS) was tuned with 10,000 warm-up steps and 100,000 samples were drawn from 4 chains that were run simultaneously. The tuning samples and the first 50,000 samples in each chain were discarded. To compute SUCRA scores, 200,000 samples

were drawn from the posterior distribution of d_i and treatments were ranked for each draw. The SUCRA score for treatment i was calculated as:

$$\text{SUCRA}_i = \frac{n-1-\langle R_i \rangle}{n-1} \tag{10}$$

where $\langle R_i \rangle$ is the mean rank for treatment i across all draws and n is the number of treatments ($R_i \in [0, n-1]$). Posterior samples were used to compute the mean and 94% credible intervals for ATE values d_i of all treatments relative to Metformin, the baseline treatment.

Supplementary Tables

Supplementary Table 1: Full summary statistics of the study population at snapshot level Baseline characteristics of the training and test sets by clinical cohort for confounding variables. The top table displays mean and standard deviation for continuous variables. The bottom table displays the proportion present for binary variables. Training and test datasets were split for each of the 10 clinical cohorts based on age, prior use of insulin and comorbidity index values. The number of available treatments varies for each cohort and is typically higher for larger cohort sizes. ZCTA: Zip Code Tabulation Area; EGFR: Estimated Glomerular Filtration Rate; ASCVD: Atherosclerotic cardiovascular disease.

Insulin Status	Non-user										User									
Cohort	A		B		C		D		E		F		G		H		I		J	
Feature	Train	Test	Train	Test	Train	Test	Train	Test	Train	Test	Train	Test	Train	Test	Train	Test	Train	Test	Train	Test
Baseline HbA1c	10.6 ± 1.4	10.6 ± 1.4	10.6 ± 1.4	10.5 ± 1.4	10.5 ± 1.4	10.6 ± 1.4	10.3 ± 1.3	10.3 ± 1.3	10.3 ± 1.3	10.3 ± 1.3	10.5 ± 1.3	10.5 ± 1.3	10.6 ± 1.3	10.6 ± 1.3	10.6 ± 1.4	10.6 ± 1.3	10.3 ± 1.2	10.2 ± 1.1	10.2 ± 1.2	10.2 ± 1.2
ZCTA Median Income	60755.1 ± 22942.4	60791.9 ± 23147.7	59166.9 ± 22118.4	58943.3 ± 22415.7	55600.5 ± 20605.6	56197.3 ± 21234.9	59038.8 ± 23523.4	59252.0 ± 24276.1	56521.9 ± 22888.7	56094.7 ± 22840.2	59089.1 ± 22695.9	58921.9 ± 22113.0	56125.1 ± 21388.8	56317.2 ± 21083.6	54583.3 ± 20424.8	53826.6 ± 20355.3	58092.5 ± 24715.7	57166.7 ± 23273.6	53894.9 ± 21548.4	54241.6 ± 21252.9
ZCTA % White	62.3 ± 22.3	62.1 ± 22.6	60.9 ± 23.6	60.4 ± 23.6	58.9 ± 23.9	59.8 ± 23.8	63.9 ± 24.4	63.3 ± 24.1	61.4 ± 25.0	63.1 ± 24.0	63.5 ± 22.9	63.4 ± 23.0	60.5 ± 23.6	60.6 ± 23.6	61.5 ± 23.2	60.8 ± 24.2	62.8 ± 24.7	64.6 ± 24.0	64.8 ± 23.6	64.0 ± 23.7
ZCTA % Native Am.	0.5 ± 0.8	0.6 ± 1.1	0.5 ± 0.6	0.6 ± 1.0	0.6 ± 0.9	0.5 ± 0.9	0.5 ± 0.5	0.5 ± 0.8	0.5 ± 0.6	0.5 ± 0.5	0.6 ± 0.9	0.6 ± 1.0	0.6 ± 0.8	0.5 ± 0.7	0.5 ± 0.8	0.6 ± 1.0	0.7 ± 3.3	0.5 ± 0.7	0.5 ± 0.6	0.5 ± 0.5
ZCTA % Black	15.8 ± 19.3	16.0 ± 19.6	16.5 ± 20.2	16.5 ± 20.3	18.5 ± 21.6	17.4 ± 20.9	15.4 ± 20.1	15.3 ± 19.8	15.8 ± 20.8	15.1 ± 20.1	17.2 ± 20.4	17.2 ± 20.4	18.3 ± 21.3	18.0 ± 21.2	17.6 ± 20.9	18.1 ± 21.5	16.2 ± 19.6	16.0 ± 19.9	15.1 ± 19.5	14.8 ± 19.3
ZCTA % Asian	7.9 ± 10.8	7.9 ± 10.9	8.2 ± 11.4	8.2 ± 11.4	8.1 ± 11.5	8.1 ± 11.5	7.7 ± 10.9	8.0 ± 11.5	7.7 ± 11.4	7.4 ± 10.9	6.6 ± 9.7	6.6 ± 9.7	6.9 ± 9.5	7.0 ± 9.8	6.9 ± 9.7	6.7 ± 9.7	7.2 ± 10.8	6.5 ± 9.8	6.3 ± 10.7	6.4 ± 10.3
Age	50.9 ± 8.8	50.9 ± 8.8	52.9 ± 8.0	53.0 ± 7.9	55.0 ± 7.1	54.9 ± 7.2	70.4 ± 5.2	70.4 ± 5.3	73.4 ± 6.4	73.3 ± 6.5	52.0 ± 8.3	52.0 ± 8.4	53.5 ± 7.5	53.5 ± 7.6	55.4 ± 6.6	55.4 ± 6.7	70.3 ± 4.7	70.3 ± 5.0	72.7 ± 6.1	72.9 ± 6.0
Creatinine Lab	0.8 ± 0.2	0.8 ± 0.2	0.9 ± 0.3	0.8 ± 0.3	1.0 ± 0.4	1.0 ± 0.4	0.9 ± 0.3	0.9 ± 0.3	1.1 ± 0.4	1.1 ± 0.5	0.8 ± 0.2	0.8 ± 0.2	0.9 ± 0.3	0.9 ± 0.3	1.1 ± 0.5	1.1 ± 0.5	1.0 ± 0.3	1.0 ± 0.3	1.2 ± 0.5	1.2 ± 0.5
EGFR Lab	102.3 ± 18.3	102.5 ± 18.3	98.5 ± 20.3	98.7 ± 20.1	87.6 ± 25.1	87.7 ± 25.4	81.0 ± 18.3	80.8 ± 18.5	69.1 ± 22.4	69.1 ± 22.6	99.4 ± 19.6	99.9 ± 19.7	96.5 ± 20.8	96.4 ± 21.2	80.4 ± 28.0	81.4 ± 28.1	78.9 ± 19.1	78.9 ± 19.5	62.8 ± 22.7	62.9 ± 23.3

Insulin Status	Non-User										User									
Cohort	A		B		C		D		E		F		G		H		I		J	
Feature	Train: % Present	Test: % Present	Train: % Present	Test: % Present	Train: % Present	Test: % Present	Train: % Present	Test: % Present	Train: % Present	Test: % Present	Train: % Present	Test: % Present	Train: % Present	Test: % Present	Train: % Present	Test: % Present	Train: % Present	Test: % Present	Train: % Present	Test: % Present
Renal Disease	0.1	0.1	4.1	4.8	40.1	41.8	4	4.1	60.3	61.5	0	0.1	3.5	4	48.3	48	4.2	5.1	66.8	70.1
Cancer	0.1	0.1	5.9	5.8	17.1	17	3.5	3	20	20.1	0	0	3.2	2.9	15.3	14.8	3	1.9	16.1	15.8
Metastatic Carcinoma	0	0	0	0	5	4.4	0	0	2.9	3.1	0	0	0	0	2	2.7	0	0	1.5	1.8
Connective Tissue Disease	0.9	0.8	2.3	2.3	6.5	6.3	1.4	1.6	4	5.1	0.7	0.8	1.4	1.8	6.8	6.8	1.5	1.7	5	4.2
Dementia	0.1	0.1	0.3	0.3	1	1.4	1.6	1.4	5.4	6.7	0	0.1	0	0.2	1.6	1.7	1.9	1.3	7.4	6.4
Paraplegia and Hemiplegia	0	0	0.5	0.5	3.8	4.4	0.2	0.1	3.7	3	0	0	0.4	0.4	4.7	4.4	0.1	0.2	2.3	3.3

Cerebrovascular Disease	1.4	1.5	5.9	6.2	22.3	23.8	6.5	6.3	28	26.6	1.7	1.9	5.6	6.2	26.4	25.4	6.4	5.9	25.8	26.5
Chronic Pulmonary Disease	9.1	8.8	17.2	18.4	38.1	36.7	11.7	11	32.7	35.2	11	10.3	18.6	17.8	42.8	43.1	12.6	13.7	38.4	34.8
Peptic Ulcer Disease	0.3	0.4	1.4	1.2	4.9	4.9	0.6	0.7	4.1	3.2	0.3	0.4	1	0.9	4	4.1	0.5	0.6	3.2	3.6
Diabetes w Complications	1.4	1.6	76.7	75.5	84.1	84.6	35	35.9	93.7	91	1.9	1.9	83.5	83.8	92	92.7	53	52.7	95.6	95.9
Diabetes w/o Complications	91.5	91.4	98.6	98.5	98.8	98.7	92.9	92.7	98.8	98.8	97.2	97	99.3	99.1	99.6	99.6	96.2	95.8	99.3	99.1
Mild Liver Disease	5.8	5.8	12.9	13.9	30.9	29.6	4.7	5.1	16.9	17.7	5.8	6.6	11.4	10.4	28.5	29.7	6.4	5.3	17.5	16.2
Severe Liver Disease	0	0	0.1	0	3.2	2.9	0	0	1.4	1.8	0	0	0.1	0	3.1	3.6	0	0	2	1.9
Obesity	34	33.4	43.5	43	53.6	51.7	25	24.6	37.3	37.1	41.3	39.7	48.9	48.8	60.3	60.4	31.5	31.7	48.2	46.7
Hypoglycemia	0.7	0.8	1.7	1.6	3.6	3.6	1.2	1.1	4.6	5.3	1.9	1.6	4.1	3.9	6.7	6.8	3	3.2	9.3	9.8
ASCVD	5.4	5.1	12.7	12.4	29.7	30.7	13.7	15.2	39.4	39.7	7.5	7.9	14	15.7	39	38.2	18.8	19.9	47.4	46.2
Peripheral Vascular Disease	1	1	6.4	6.3	21.2	22.4	5.9	5.9	31.6	28.4	1.4	1.4	6.2	7.3	26	26.2	9	8.4	31.4	31.1
Heart Failure	3.3	3	9.4	9.4	28.6	27.4	6.4	7.1	32.5	34	4.4	4.4	11.3	11.3	38.2	36.3	7.5	10.3	43.7	41.2
End-Stage Renal Disease	0	0	0.1	0.2	1.9	2.1	0.1	0.1	1.8	2.7	0	0	0.2	0.1	3.5	3.5	0.1	0.1	4.3	3.4
Dialysis	0	0	0.2	0.1	0.9	0.8	0.3	0.1	0.6	0.6	0	0	0	0.1	1.8	1.5	0.3	0	0.9	1.3
Chronic Kidney Disease	0.3	0.5	6.3	7.4	41.7	42.7	7.7	8.1	63.2	63.9	0.6	0.5	6.7	7.6	50.6	50.4	8.9	10.2	69.4	72.6
Fructosamine Test	0.5	0.6	0.9	1	1.6	1.1	0.4	0.9	0.3	1.2	0.6	1	1.3	1.4	1.2	1.8	1.5	1.6	1.9	2.2
Gastroparesis	0.1	0.1	0.6	0.6	1.2	1.1	0.1	0.2	0.7	0.3	0.3	0.2	1.1	0.9	1.6	1.9	0.7	0.3	1.3	1.1

Supplementary Table 2: Real-world Observations of cohort Treatment Ranking Concordance

Concordant percent values indicate the percent of each group that received a drug recommended for their cohort divided into rank 1-3, 4-10 or 11-n. One percent of patients in Cohort A were on a highly ranked treatment, seven percent were on a middle-ranked treatment, ninety-two were on a low ranked treatment.

Cohort Subgroup	Number of Snapshots	Number of Treatments	% Concordant Top 3	% Concordant Top 4-10	% Concordant (>10 or none)
A	43532	69	1.0%	6.8%	92.2%
B	17072	50	1.5%	9.6%	88.9%
C	7331	37	3.6%	7.3%	89.1%
D	7177	30	2.6%	11.3%	86.1%
E	3471	18	9.9%	37.3%	52.8%
F	10734	43	1.8%	6.6%	91.6%
G	10447	43	1.5%	6.8%	91.7%
H	7180	35	4.4%	9.0%	86.6%
I	2931	15	8.5%	31.2%	60.3%
J	3425	19	10.3%	23.8%	65.9%

Supplementary Table 3: Summary statistics for train/test clinical cohorts data imputations.

	Train	Test	Imputed Mean Value
Number of snapshots	113300	28325	NA
#Snapshots w/ missing age	0	0	-
#Snapshots w/ missing gender	0	0	-
#Snapshots w/ missing egfr_val_mL/min/1.73m2	5583	1378	94.63
#Snapshots w/ missing creatinine_lab	4882	1192	0.88
#Snapshots w/ missing median_income	3277	797	58651.67

#Snapshots w/ missing p_amrind_and_alskntv	3135	756	0.54
#Snapshots w/ missing p_white	3135	756	61.83
#Snapshots w/ missing p_black_or_aframr	3135	756	16.50
#Snapshots w/ missing p_asian	3135	756	7.58

Supplementary Table 4. Causal modeling derived treatment strategy rankings by clinical cohorts. Treatment regimens are ranked according to their SUCRA scores for each clinical cohort. A-E clinical cohorts did not contain Insulin as a prior treatment. F-J clinical cohorts contained Insulin as a prior treatment. Abbreviations used: INS= Insulin, GLP-1 = Glucagon-Like Peptide-1 Receptor Agonist; SULF: Sulfonylureas; METF = Metformin; MEGL: Meglitinide; DPP-4 = Dipeptidyl Peptidase 4 Inhibitor; TZD = Thiazolidinedione; SGLT2 = Sodium-Glucose Transport Protein 2 Inhibitor; AGI = Alpha-Glucosidase Inhibitor.

	Insulin Non-user Clinical Cohorts					Insulin User Clinical Cohorts				
Treatment Rank	A	B	C	D	E	F	G	H	I	J
1	METF + SGLT2 + TZD	DPP-4 + METF + SGLT2 + SULF + TZD	GLP-1 + METF	BASAL INS + DPP-4 + METF	DPP-4 + METF + SULF	BASAL INS + GLP-1 + METF + SGLT2	BASAL INS + GLP-1 + SGLT2	BASAL INS + GLP-1 + METF + SGLT2	BASAL INS + GLP-1 + METF	BASAL INS + GLP-1 + METF
2	GLP-1 + METF + SGLT2	GLP-1 + METF + SGLT2	GLP-1 + METF + SGLT2 + SULF	SULF + TZD	METF + TZD	BASAL INS + GLP-1 + SGLT2	BASAL INS + DPP-4 + METF + SGLT2	BASAL INS + BOLUS INS + GLP-1 + METF	METF + MIXED INS	METF
3	BASAL INS + DPP-4 + METF + SGLT2	GLP-1 + SGLT2	METF + SGLT2	DPP-4 + METF + SGLT2	METF + SULF + TZD	BASAL INS + DPP-4 + METF + SGLT2	BASAL INS + GLP-1 + METF + SGLT2	BASAL INS + GLP-1 + METF	BASAL INS + DPP-4 + METF	BASAL INS + METF
4	DPP-4 + SGLT2	DPP-4 + METF + SGLT2	GLP-1 + METF + SGLT2	DPP-4 + METF + TZD	METF + SGLT2 + SULF	BASAL INS + BOLUS INS + GLP-1 + METF	BASAL INS + GLP-1 + METF	BASAL INS + METF + SGLT2	BASAL INS + BOLUS INS + METF	BASAL INS + BOLUS INS + DPP-4
5	METF + SGLT2	GLP-1 + METF + SGLT2 + SULF	BASAL INS + GLP-1 + METF	BASAL INS + DPP-4 + METF + SULF	BASAL INS + METF + SULF	BASAL INS + METF + SGLT2 + SULF	BASAL INS + DPP-4 + GLP-1 + METF	BASAL INS + BOLUS INS + METF + SGLT2	BASAL INS + METF + SULF	BASAL INS + BOLUS INS + GLP-1

6	DPP-4 + METF + SGLT2 + SULF + TZD	DPP-4 + METF + SGLT2 + SULF	GLP-1 + METF + SULF	GLP-1 + METF	METF + SULF	DPP-4 + METF	BASAL INS + GLP-1 + SULF	BASAL INS + GLP-1 + METF + SULF	BASAL INS + METF	BASAL INS + DPP-4 + METF
7	DPP-4 + METF + SGLT2	GLP-1 + METF	METF + SGLT2 + SULF	GLP-1 + METF + SULF	DPP-4 + METF	BASAL INS + BOLUS INS + GLP-1	BASAL INS + BOLUS INS + SGLT2	BASAL INS + BOLUS INS + GLP-1	BASAL INS + GLP-1	BASAL INS + DPP-4
8	GLP-1 + METF	METF + SGLT2	METF + TZD	METF + TZD	BASAL INS + DPP-4 + SULF	GLP-1 + METF	BASAL INS + METF + SGLT2 + SULF	BASAL INS + DPP-4	METF	BASAL INS + BOLUS INS + METF + SULF
9	BASAL INS + DPP-4 + GLP-1 + METF	BASAL INS + GLP-1	DPP-4 + METF + SULF + TZD	DPP-4 + METF	SULF + TZD	BASAL INS + GLP-1 + METF	BASAL INS + GLP-1 + METF + SULF	BASAL INS + DPP-4 + METF + SULF	BASAL INS + DPP-4 + METF + SULF	BASAL INS + BOLUS INS
10	BASAL INS + GLP-1 + METF	GLP-1 + METF + SULF	GLP-1 + SULF	METF + SGLT2 + SULF	METF	BASAL INS + BOLUS INS + METF + SGLT2	BASAL INS + GLP-1	BASAL INS + BOLUS INS + METF + SULF	METF + SULF	BASAL INS + BOLUS INS + METF

Supplementary Table 5: Treatment ranks for each clinical cohort (ranking based on SUCRA) and associated average rank positions (from model posterior distributions) and 95% confidence internals. Average rank positions were estimated from 200000 random samples of ATE posterior distributions (as estimated in NMA model) where in each sample treatments were ranked/ordered in ascending order of ATEs. Credible intervals associated with ranks were computed from standard errors of the mean/average times 1.96.

cohort	treatment	sucra	rank	avg_rank_position	± rank_position_conf_95
A	METF + SGLT2 + TZD	0.98	1	1.48	<0.01
A	GLP-1 + METF + SGLT2	0.97	2	2.24	<0.01
A	BASAL INS + DPP-4 + METF + SGLT2	0.96	3	2.28	<0.01

A	DPP-4 + SGLT2	0.94	4	4.34	<0.01
A	METF + SGLT2	0.92	5	5.32	<0.01
A	DPP-4 + METF + SGLT2 + SULF + TZD	0.92	6	5.41	<0.01
A	DPP-4 + METF + SGLT	0.89	7	7.18	<0.01
A	GLP-1 + METF	0.88	8	9.93	<0.01
A	BASAL INS + DPP-4 + GLP-1 + METF	0.85	9	10.25	<0.01
A	BASAL INS + GLP-1 + METF	0.84	10	10.65	<0.01
B	DPP-4 + METF + SGLT2 + SULF + TZD	0.98	1	1.05	<0.01
B	GLP-1 + METF + SGLT2	0.95	2	2.45	<0.01
B	GLP-1 + SGLT2	0.95	3	2.49	<0.01
B	DPP-4 + METF + SGLT2	0.91	4	4.19	<0.01
B	GLP-1 + METF + SGLT2 + SULF	0.90	5	4.81	<0.01
B	DPP-4 + METF + SGLT2 + SULF	0.87	6	6.51	<0.01
B	GLP-1 + METF	0.86	7	6.54	<0.01
B	METF + SGLT2	0.82	8	8.68	<0.01
B	BASAL INS + GLP-1	0.79	9	9.98	<0.01
B	GLP-1 + METF + SULF	0.79	10	10.07	<0.01

C	GLP-1 + METF	0.97	1	1.18	<0.01
C	GLP-1 + METF + SGLT2 + SULF	0.92	2	2.7	<0.01
C	METF + SGLT2	0.92	3	2.72	<0.01
C	GLP-1 + METF + SGLT2	0.87	4	4.74	<0.01
C	BASAL INS + GLP-1 + METF	0.86	5	5.03	<0.01
C	GLP-1 + METF + SULF	0.79	6	7.32	<0.01
C	METF + SGLT2 + SULF	0.78	7	7.97	<0.01
C	METF + TZD	0.77	8	8.26	<0.01
C	DPP-4 + METF + SULF + TZD	0.76	9	8.41	0.01
C	GLP-1 + SULF	0.71	10	10.35	0.01
D	BASAL INS + DPP-4 + METF	0.96	1	1.12	<0.01
D	SULF + TZD	0.89	2	3.07	<0.01
D	DPP-4 + METF + SGLT2	0.87	3	3.70	<0.01
D	DPP-4 + METF + TZD	0.84	4	4.79	<0.01
D	BASAL INS + DPP-4 + METF + SULF	0.82	5	5.22	0.01
D	GLP-1 + METF	0.81	6	5.35	0.01
D	GLP-1 + METF + SULF	0.74	7	7.46	<0.01
D	METF + TZD	0.68	8	9.21	0.01
D	DPP-4 + METF	0.67	9	9.57	<0.01

D	METF + SGLT2 + SULF	0.66	10	9.88	<0.01
E	DPP-4 + METF + SULF	0.85	1	2.54	<0.01
E	METF + TZD	0.73	2	4.63	0.01
E	METF + SULF + TZD	0.71	3	4.8	0.01
E	METF + SGLT2 + SULF	0.67	4	5.51	0.02
E	BASAL INS + METF + SULF	0.67	5	5.67	0.01
E	METF + SULF	0.66	6	5.75	<0.01
E	DPP-4 + METF	0.65	7	5.85	0.01
E	BASAL INS + METF + SULF	0.53	8	7.92	0.01
E	SULF + TZD	0.52	9	8.09	0.01
E	METF	0.44	10	9.47	<0.01
F	BASAL INS + GLP-1 + METF + SGLT2	0.96	1	1.56	<0.01
F	BASAL INS + GLP-1 + SGLT2	0.96	2	1.57	<0.01
F	BASAL INS + DPP-4 + METF + SGLT2	0.91	3	3.71	<0.01
F	BASAL INS + BOLUS INS + GLP-1 + METF	0.91	4	3.75	<0.01
F	BASAL INS + METF + SGLT2 + SULF	0.86	5	5.73	<0.01
F	DPP-4 + METF	0.84	6	6.62	<0.01

F	BASAL INS + BOLUS INS + GLP-1	0.83	7	7.21	<0.01
F	GLP-1 + METF	0.83	8	7.24	<0.01
F	BASAL INS + GLP-1 + METF	0.81	9	7.82	<0.01
F	BASAL INS + BOLUS INS + METF + SGLT2	0.75	10	10.5	<0.01
G	BASAL INS + GLP-1 + SGLT2	0.95	1	2.08	0.01
G	BASAL INS + DPP-4 + METF + SGLT2	0.94	2	2.45	<0.01
G	BASAL INS + GLP-1 + METF + SGLT2	0.93	3	3.11	<0.01
G	BASAL INS + GLP-1 + METF	0.89	4	4.24	<0.01
G	BASAL INS + DPP-4 + GLP-1 + METF	0.86	5	6.03	<0.01
G	BASAL INS + GLP-1 + SULF	0.85	6	6.32	0.02
G	BASAL INS + BOLUS INS + SGLT2	0.84	7	6.61	<0.01
G	BASAL INS + METF + SGLT2 + SULF	0.81	8	7.59	0.01
G	BASAL INS + GLP-1 + METF + SULF	0.80	9	8.19	<0.01

G	BASAL INS + GLP-1	0.75	10	10.12	<0.01
H	BASAL INS + GLP-1 + METF + SGLT2	0.94	1	1.91	<0.01
H	BASAL INS + BOLUS INS + GLP-1 + METF	0.91	2	3.03	<0.01
H	BASAL INS + GLP-1 + METF	0.90	3	3.06	<0.01
H	BASAL INS + METF + SGLT2	0.87	4	4.35	<0.01
H	BASAL INS + BOLUS INS + METF + SGLT2	0.85	5	5.07	<0.01
H	BASAL INS + GLP-1 + METF + SULF	0.84	6	5.24	<0.01
H	BASAL INS + BOLUS INS + GLP-1	0.80	7	6.71	<0.01
H	BASAL INS + DPP-4	0.73	8	9.22	<0.01
H	BASAL INS + DPP-4 + METF + SULF	0.67	9	11.12	0.01
H	BASAL INS + BOLUS INS + METF + SULF	0.66	10	11.34	0.01
I	BASAL INS + GLP-1 + METF	0.93	1	1.00	<0.01
I	METF + MIXED INS	0.83	2	2.48	<0.01
I	BASAL INS + DPP-4 + METF	0.80	3	2.76	<0.01

I	BASAL INS + BOLUS INS + METF	0.60	4	5.61	<0.01
I	BASAL INS + METF + SULF	0.59	5	5.71	<0.01
I	BASAL INS + METF	0.55	6	6.28	<0.01
I	BASAL INS + GLP-1	0.53	7	6.61	0.01
I	METF	0.45	8	7.62	<0.01
I	BASAL INS + DPP-4 + METF + SULF	0.43	9	7.91	0.01
I	METF + SULF	0.29	10	9.83	<0.01
J	BASAL INS + GLP-1 + METF	0.93	1	1.29	<0.01
J	METF	0.86	2	2.46	<0.01
J	BASAL INS + METF	0.71	3	5.24	<0.01
J	BASAL INS + BOLUS INS + DPP-4	0.67	4	5.88	0.01
J	BASAL INS + BOLUS INS + GLP-1	0.64	5	6.53	0.01
J	BASAL INS + DPP-4 + METF	0.61	6	6.93	0.01
J	BASAL INS + DPP-4	0.60	7	7.19	0.01
J	BASAL INS + BOLUS INS + METF + SULF	0.59	8	7.26	0.02
J	BASAL INS + BOLUS INS	0.49	9	9.01	<0.01

J	BASAL INS + BOLUS INS + METF	0.49	10	9.05	<0.01

Supplementary Figures

Supplementary Figure 1: Confounder bias adjustment with BCAUS**.** For each covariate, box plots show standardized differences between case and comparator arms for all n = 15,198 comparisons. (a) Standardized differences without adjustment and (b) after adjustment using Inverse Probability Weights output by BCAUS propensity score model. Whiskers represent 5th and 95th percentiles, boxes represent 25th-75t percentiles, and center lines represent 50th percentile.

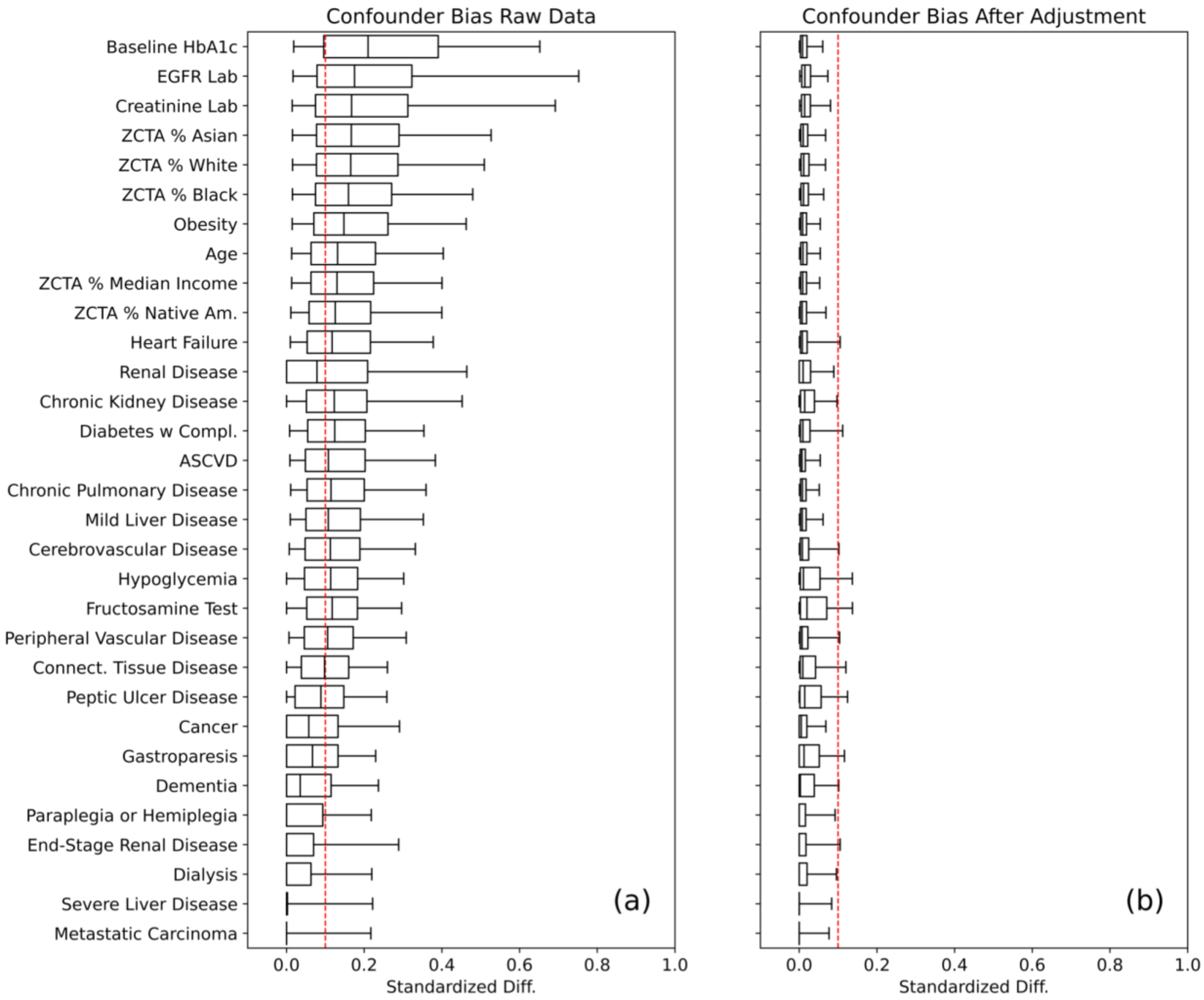

Supplementary Figure 2: Forest plot results of NMA for clinical cohort A. Average treatment effect (orange diamond) of medication combinations for insulin naïve, under 65 and CCI<=2 stratum. Blue dashed line at ATE=0. Treatment cohort size is annotated (sz). Error bars represent 95% credible intervals.

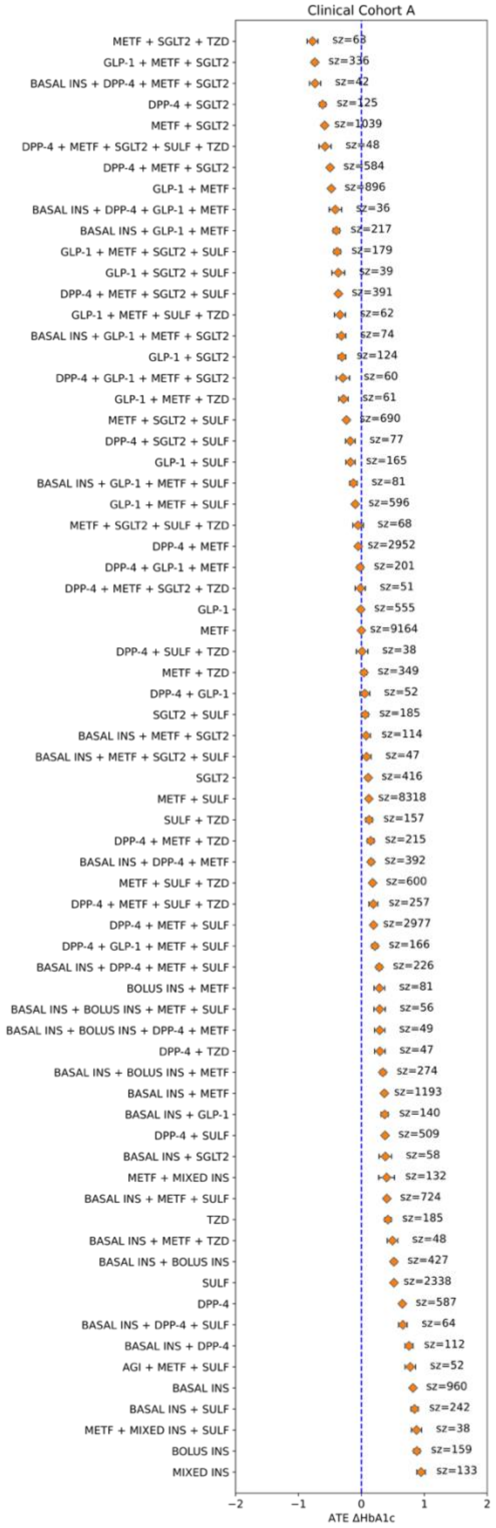

Supplementary Figure 3. Forest plot results of NMA for clinical cohort B. Average treatment effect (orange diamond) of medication combinations for insulin naïve, under 65 and 2<CCI<5 stratum. Blue dashed line at ATE=0. Treatment cohort size is annotated (sz). Error bars represent 95% credible intervals

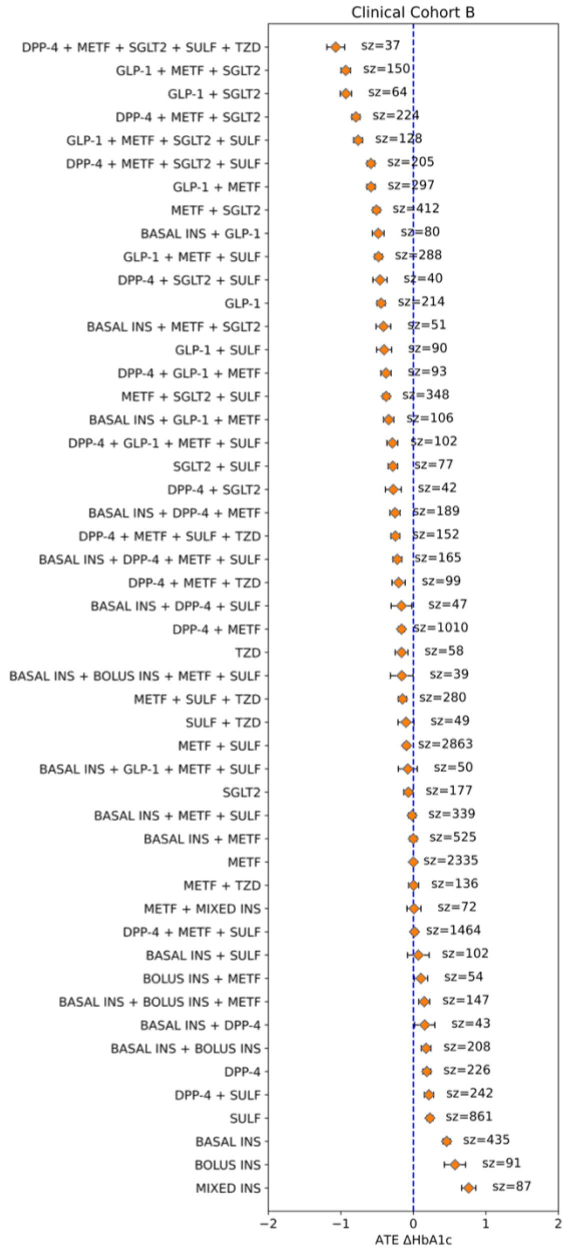

Supplementary Figure 4: Forest plot results of NMA for clinical cohort C. Average treatment effect (orange diamond) of medication combinations for insulin naïve, under 65 and CCI>=5 stratum. Blue dashed line at ATE=0. Treatment cohort size is annotated (sz). Error bars represent

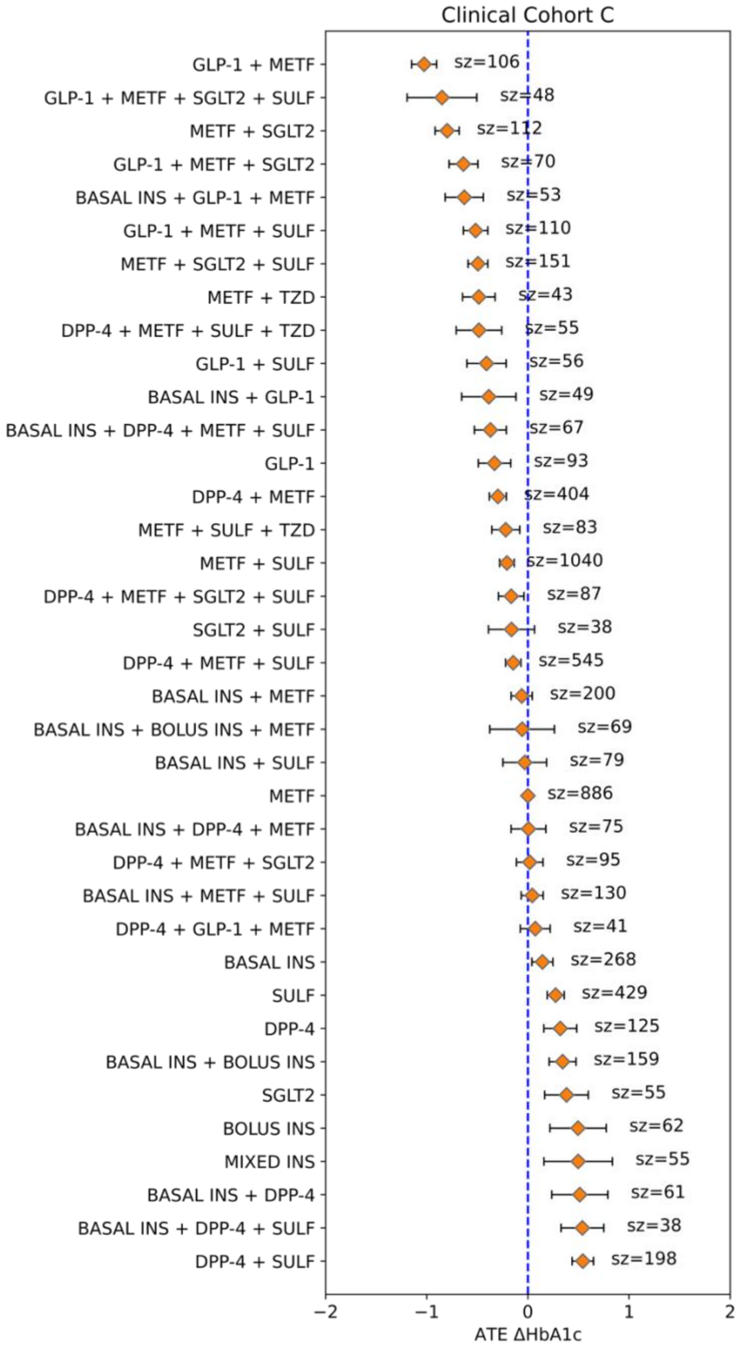

95% credible intervals.

Supplementary Figure 5: Forest plot results of NMA for clinical cohort D. Average treatment effect (orange diamond) of medication combinations for insulin naïve, over 65 and CCI<5 stratum. Blue dashed line at ATE=0. Treatment cohort size is annotated (sz). Error bars represent 95% credible intervals.

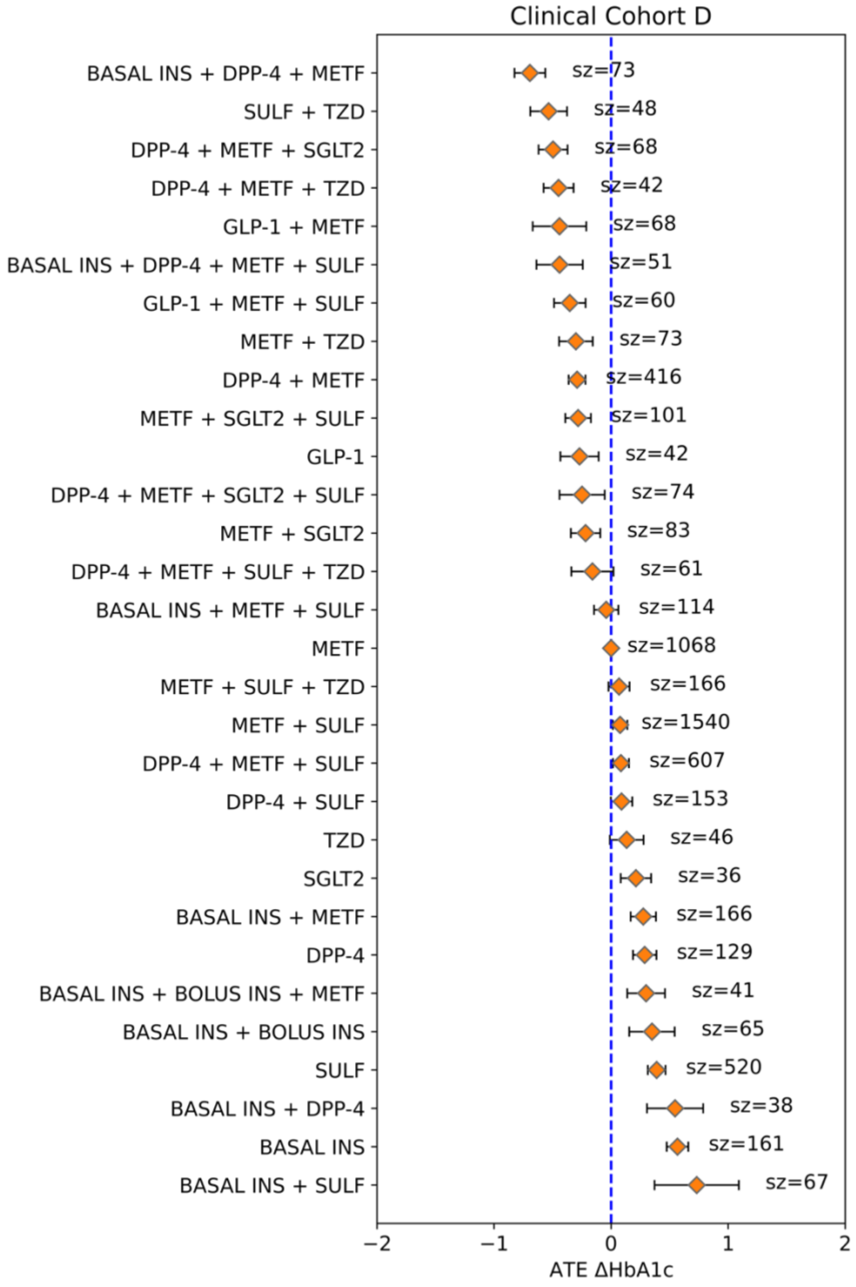

Supplementary Figure 6: Forest plot results of NMA for clinical cohort E. Average treatment effect (orange diamond) of medication combinations for insulin naïve, over 65 and CCI>=5 stratum. Blue dashed line at ATE=0. Treatment cohort size is annotated (sz). Error bars represent 95% credible intervals

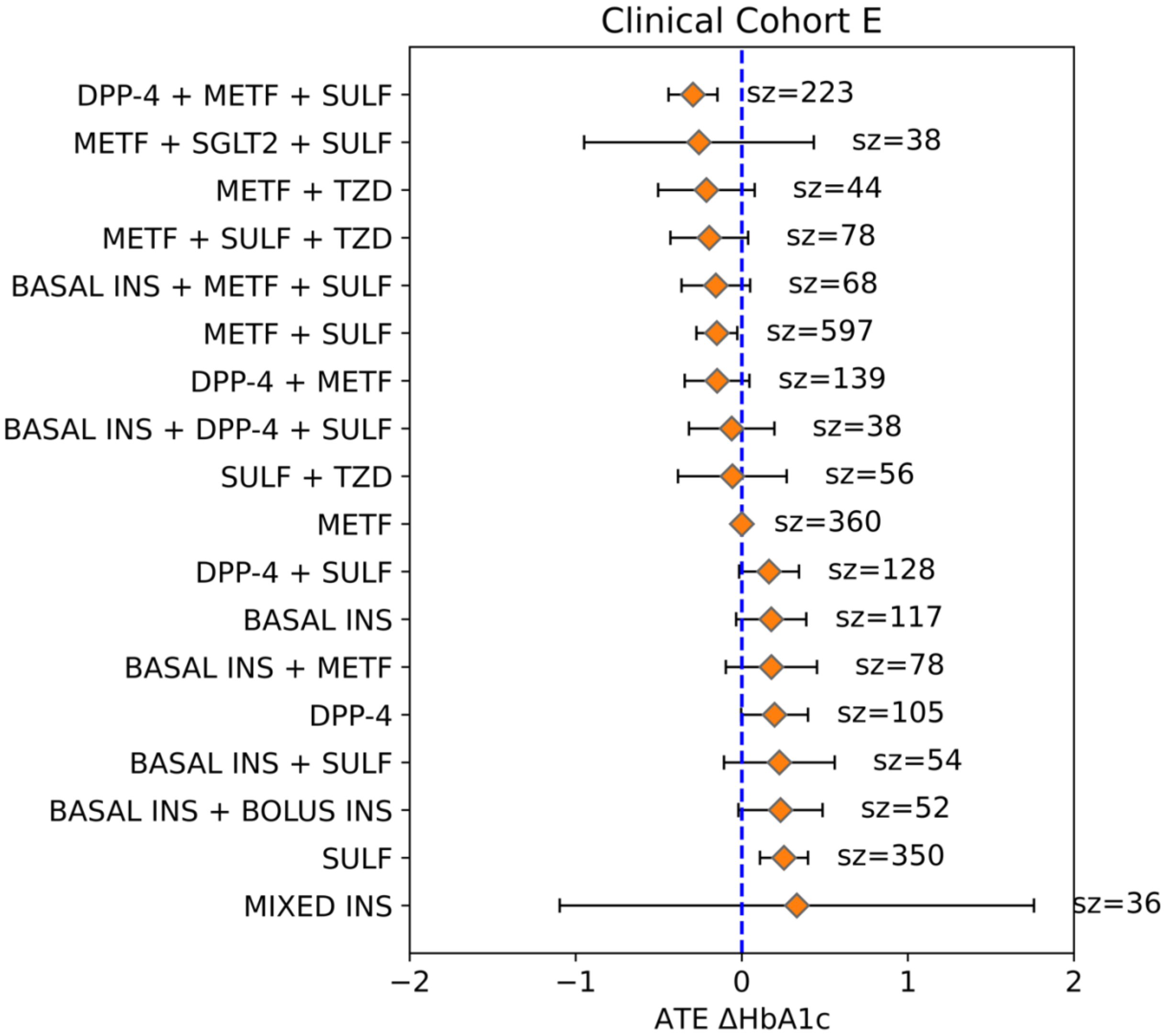

Supplementary Figure 7: Forest plot results of NMA for clinical cohort F. Average treatment effect (orange diamond) of medication combinations for insulin dependent, under 65 and CCI<=2 stratum. Blue dashed line at ATE=0. Treatment cohort size is annotated (sz). Error bars represent 95% credible intervals.

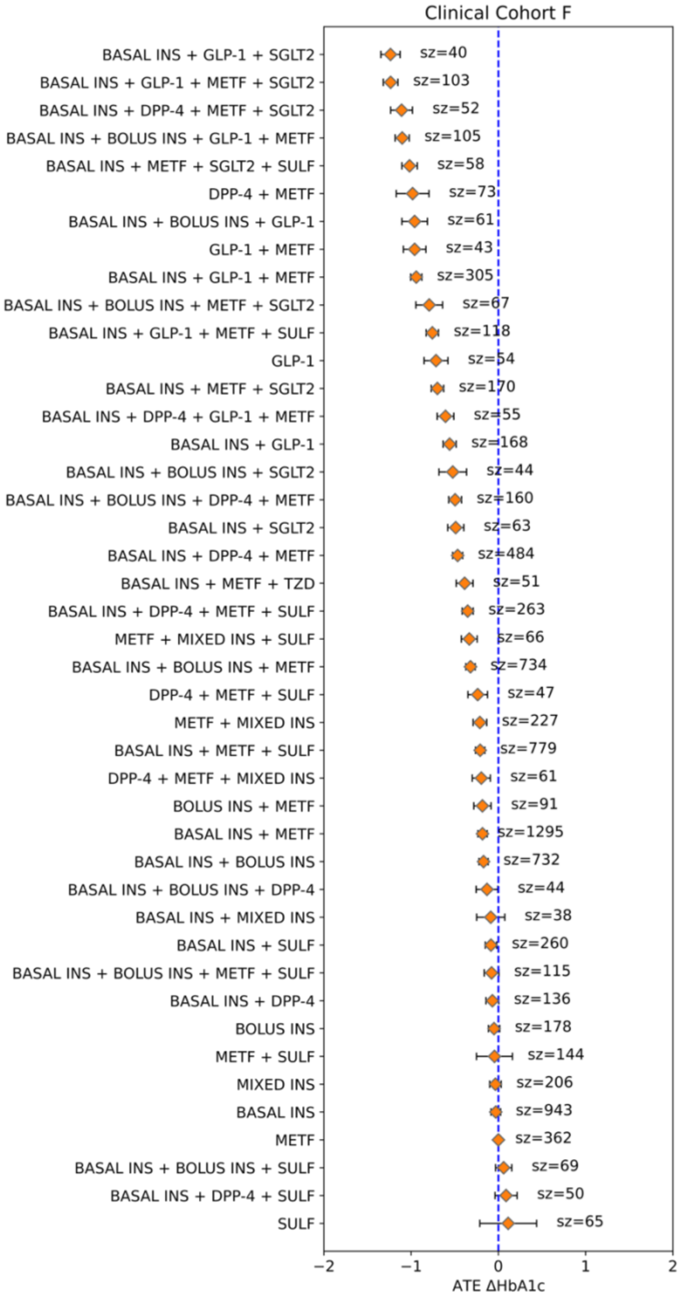

Supplementary Figure 8: Forest plot results of NMA for clinical cohort G. Average treatment effect (orange diamond) of medication combinations for insulin dependent, over 65 and 2<CCI<5 stratum. Blue dashed line at ATE=0. Treatment cohort size is annotated (sz). Error bars represent 95% credible intervals.

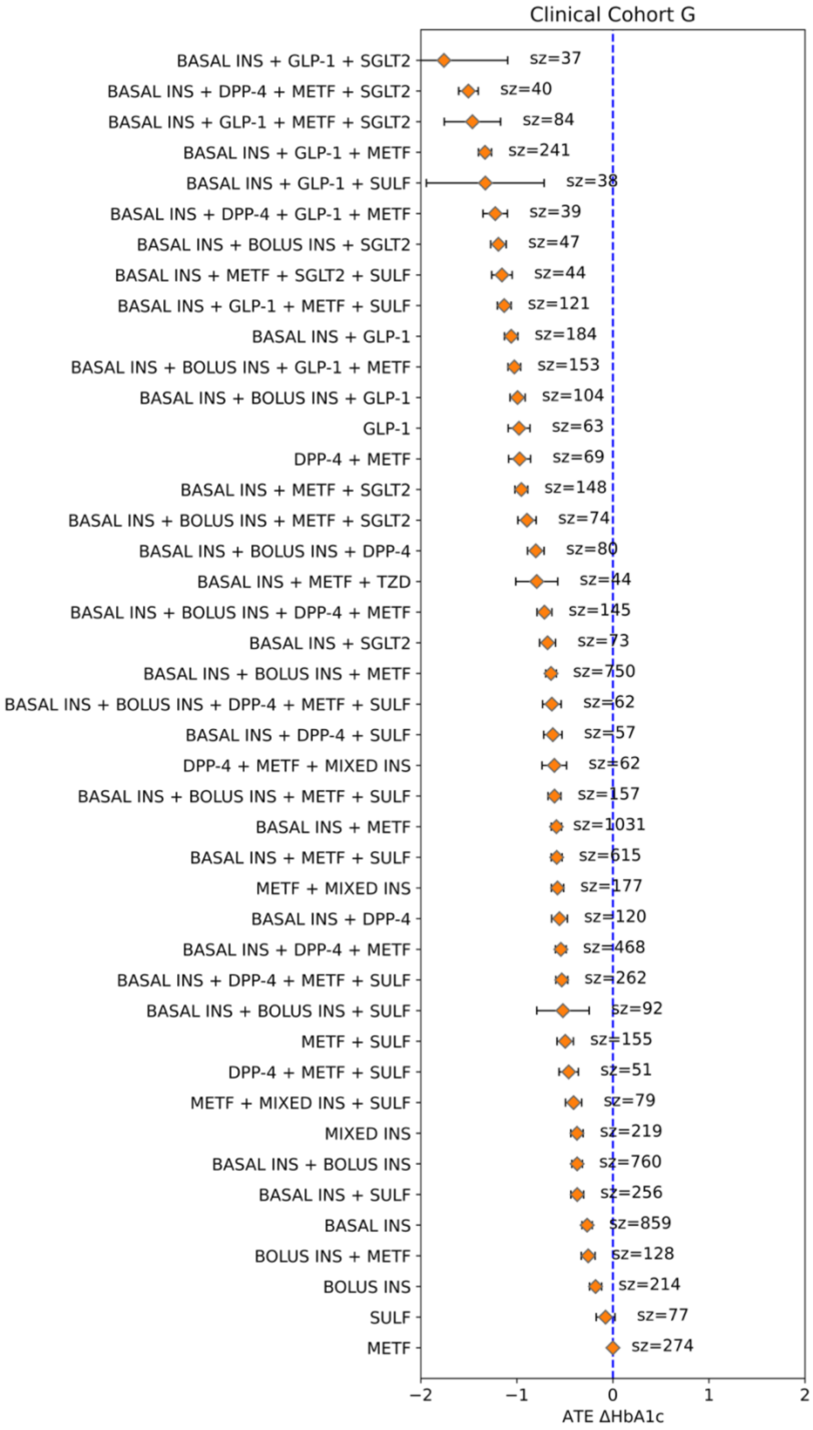

Supplementary Figure 9: Forest plot results of NMA for clinical cohort H. Average treatment effect (orange diamond) of medication combinations for insulin dependent, under 65 and CCI>5 stratum. Blue dashed line at ATE=0. Treatment cohort size is annotated (sz). Error bars represent 95% credible intervals.

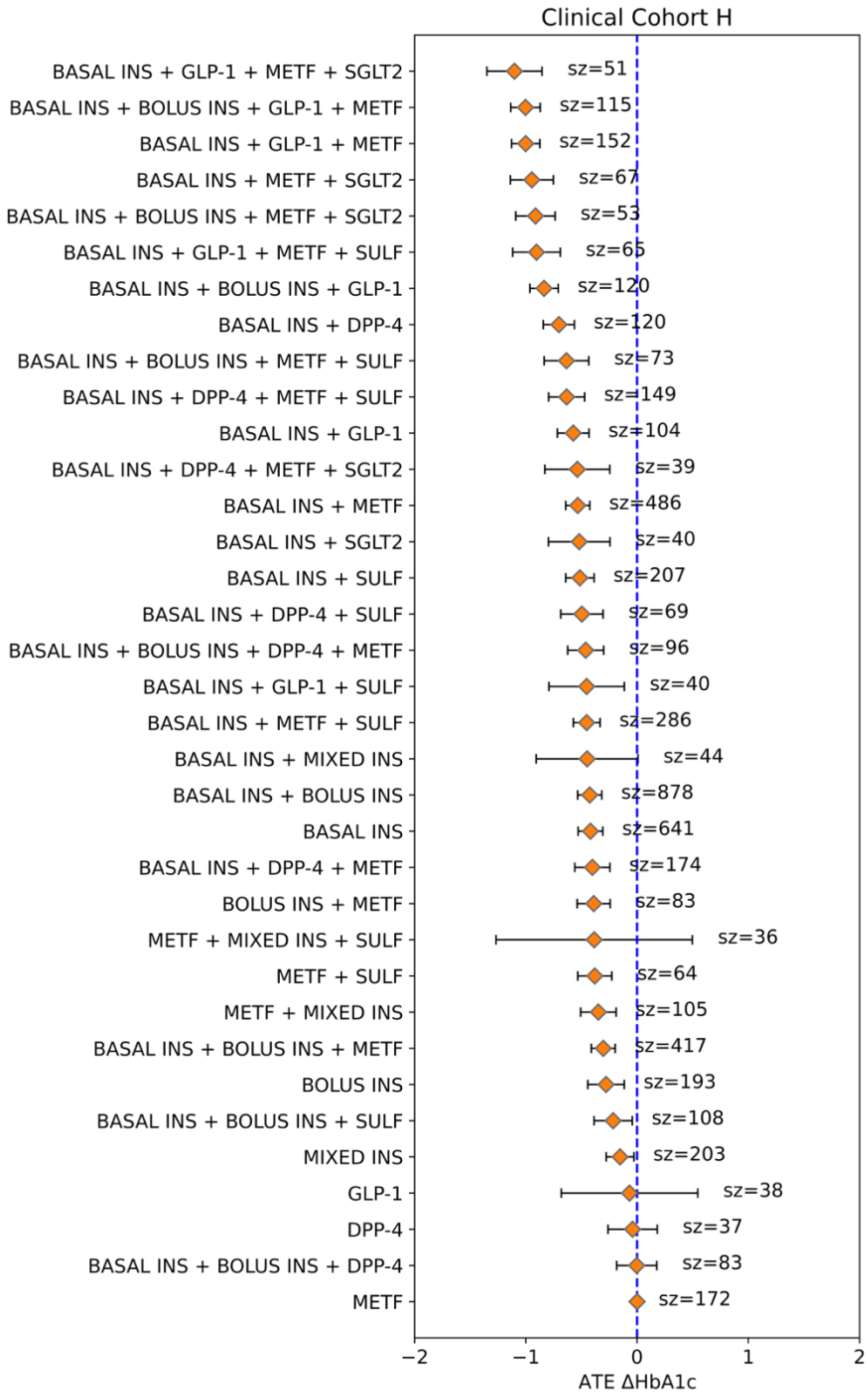

Supplementary Figure 10: Forest plot results of NMA for clinical cohort I. Average treatment effect (orange diamond) of medication combinations for insulin dependent, over65 and CCI<5 stratum. Blue dashed line at ATE=0. Treatment cohort size is annotated (sz). Error bars represent 95% credible intervals.

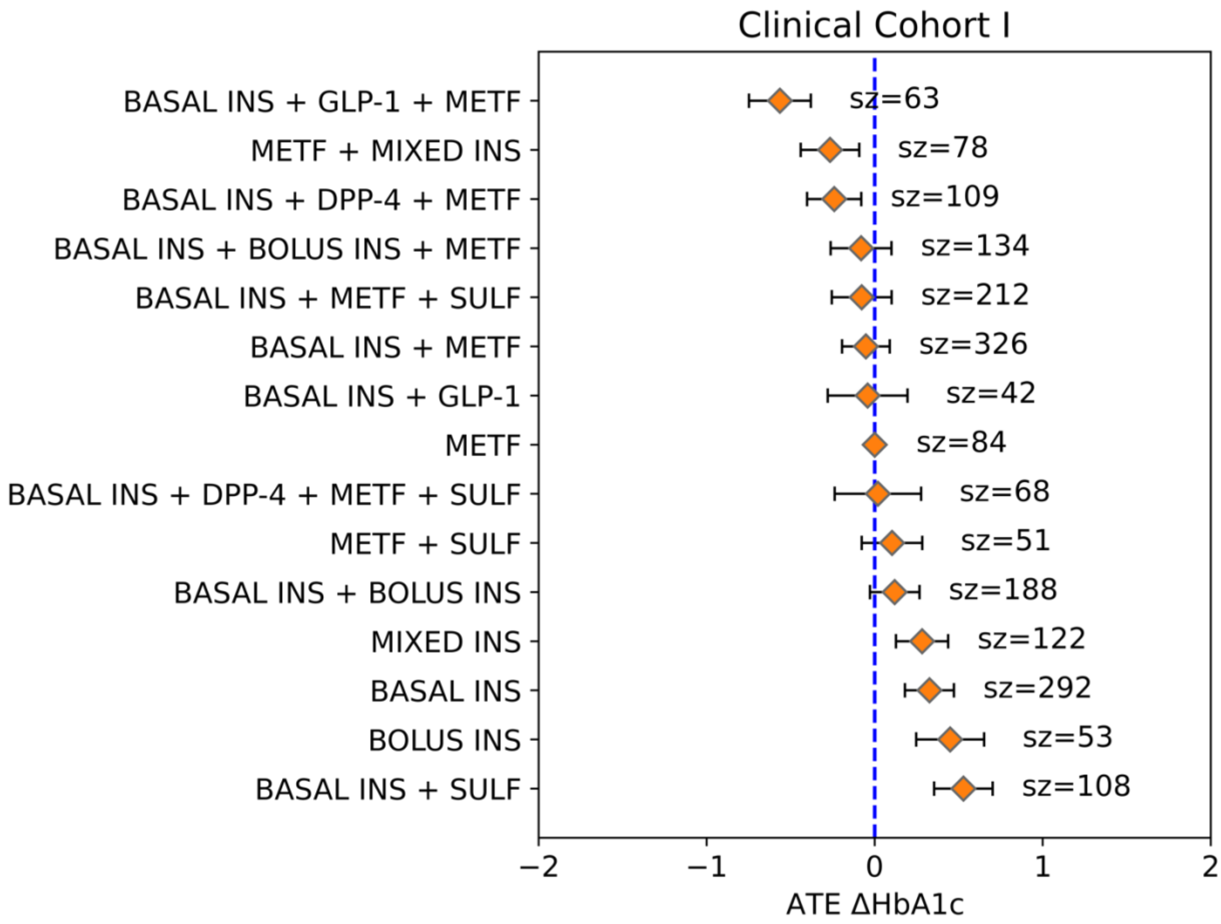

Supplementary Figure 11: Forest plot results of NMA for clinical cohort J. Average treatment effect (orange diamond) of medication combinations for insulin dependent, over 65 and CCI>5 stratum. Blue dashed line at ATE=0. Treatment cohort size is annotated (sz). Error bars represent 95% credible intervals.

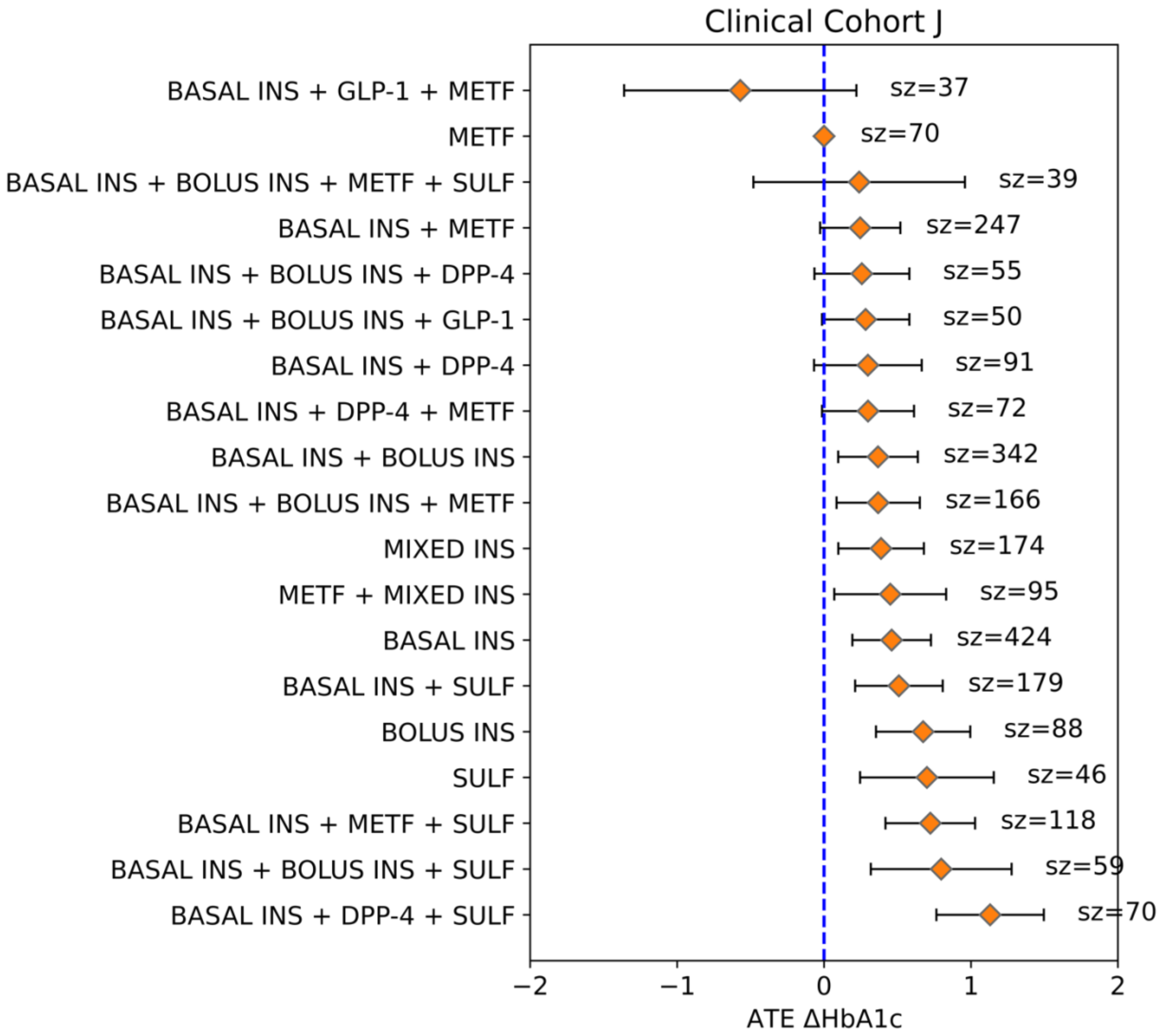

Supplementary Figure 12: Comprehensive Ranking Results of Causal Effect on Blood Sugar Reduction Attributable to Each Observed Treatment Strategy for Each Clinical Subgroup. Treatments ranked according to their SUCRA scores for each clinical cohort. Left panel shows Clinical Cohorts where prior treatment did not contain Insulin. Right panel shows Clinical Cohorts where prior treatment contained Insulin. Lower right table shows cohort definitions. Colors represent the rank of a treatment regimen in group A (left panel) or group F (right panel). Abbreviations used: INS= Insulin, GLP-1 = Glucagon-Like Peptide-1 Receptor Agonist; SULF: Sulfonylureas; METF = Metformin; MEGL: Meglitinide; DPP-4 = Dipeptidyl Peptidase 4 Inhibitor; TZD = Thiazolidinedione; SGLT2 = Sodium-Glucose Transport Protein 2 Inhibitor; AGI = Alpha-Glucosidase Inhibitor; CCI = (unweighted) Charlson Comorbidity Index

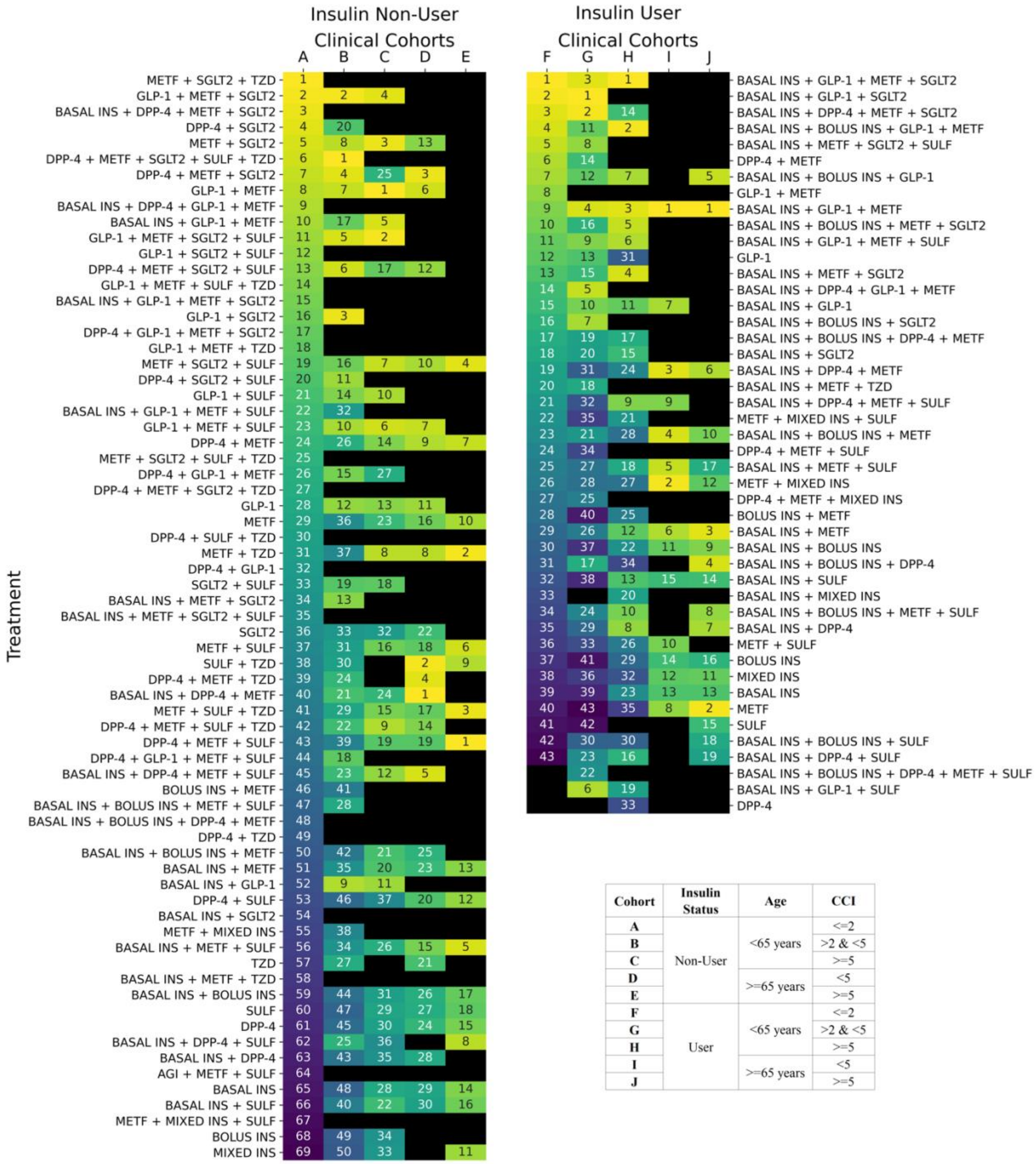

Cohort	Insulin Status	Age	CCI
A	Non-User	<65 years	<=2
B			>2 & <5
C			>=5
D		>=65 years	<5
E			>=5
F	User	<65 years	<=2
G			>2 & <5
H			>=5
I		>=65 years	<5
J			>=5

Supplementary Figure 13: Sensitivity analysis of High, Middle, and Low Ranked Treatment Strategies. Average treatment effect comparing treatments ranked 1-3 vs those ranked 11 or below (top vs middle) and treatments ranked 4-10 vs 11 or below (middle vs bottom). Each panel represents the outcome for a single clinical cohort. Error bars represent 95% confidence intervals.

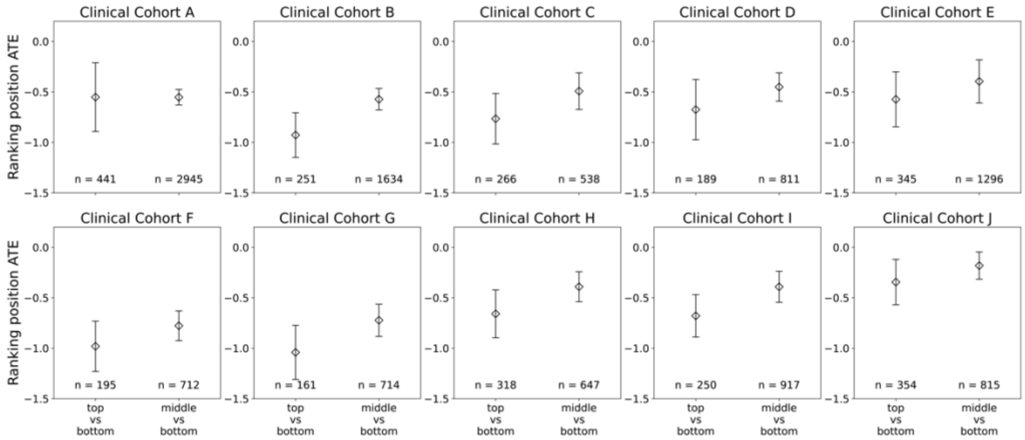

Correlations between rankings:

Each of the clinical cohorts considered in our study has a different set of ranked treatments. In order to examine correlations between rankings across different cohorts, we computed Kendall's tau pairwise between cohorts belonging to the insulin-user and insulin non-user groups separately. In each group, the top-10 treatments were chosen for every cohort and the set union of treatments seen among the top-10 for all cohorts was determined. For each cohort, all treatments that were in the set union but not in the top-10 were appended to the end of the ranked list and assigned rank 11. This procedure ensured that each cohort in the group had the same set of treatments. Kendall's tau (and associated p-value) was computed pairwise between the cohorts. As shown in Fig. S24, the tau coefficient ranged from -0.41 and 0.27 in the insulin non-user group and between -0.31and 0.35 in the insulin user group.

Supplementary Figure 14: Kendall's tau computed between rankings of cohorts: Kendall's tau computed pairwise between rankings in cohorts. Two-sided P-values are shown in parenthesis.

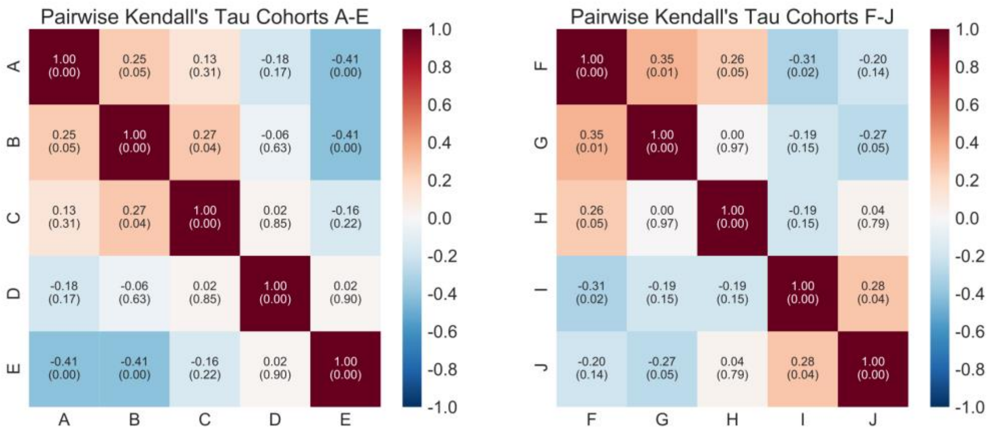

References

1 Belthangady, C., Stedden, W. & Norgeot, B. Minimizing bias in massive multi-arm observational studies with BCAUS: balancing covariates automatically using supervision. *BMC Med Res Methodol* **21**, 190, doi:10.1186/s12874-021-01383-x (2021).

2 Austin, P. C. & Stuart, E. A, Moving towards best practice when using inverse probability of treatment weighting (IPTW) using the propensity score to estimate causal treatment effects in observational studies. Statist. Med., 34 3661–3679 (2015).
