# Supplementary material for: Causal deep learning reveals the comparative effectiveness of antihyperglycemic treatments in poorly controlled diabetes
Source: Nat Commun. 2022 Nov 14;13:6921. doi: 10.1038/s41467-022-33732-9 (PMC9663714; doi:10.1038/s41467-022-33732-9)
Supplement: Supplementary file 2 — Reporting Summary [file 41467_2022_33732_MOESM2_ESM.pdf]

nature portfolio

Corresponding author(s): Beau Norgeot, PhD

Last updated by author(s): Sep 14, 2022

Reporting Summary

Nature Portfolio wishes to improve the reproducibility of the work that we publish. This form provides structure for consistency and transparency in reporting. For further information on Nature Portfolio policies, see our Editorial Policies and the Editorial Policy Checklist.

Statistics

For all statistical analyses, confirm that the following items are present in the figure legend, table legend, main text, or Methods section.

n/a	Confirmed	
☐	☒	The exact sample size (*n*) for each experimental group/condition, given as a discrete number and unit of measurement
☐	☒	A statement on whether measurements were taken from distinct samples or whether the same sample was measured repeatedly
☐	☒	The statistical test(s) used AND whether they are one- or two-sided<br>*Only common tests should be described solely by name; describe more complex techniques in the Methods section.*
☐	☒	A description of all covariates tested
☐	☒	A description of any assumptions or corrections, such as tests of normality and adjustment for multiple comparisons
☐	☒	A full description of the statistical parameters including central tendency (e.g. means) or other basic estimates (e.g. regression coefficient) AND variation (e.g. standard deviation) or associated estimates of uncertainty (e.g. confidence intervals)
☒	☐	For null hypothesis testing, the test statistic (e.g. *F*, *t*, *r*) with confidence intervals, effect sizes, degrees of freedom and *P* value noted<br>*Give P values as exact values whenever suitable.*
☐	☒	For Bayesian analysis, information on the choice of priors and Markov chain Monte Carlo settings
☒	☐	For hierarchical and complex designs, identification of the appropriate level for tests and full reporting of outcomes
☐	☒	Estimates of effect sizes (e.g. Cohen's *d*, Pearson's *r*), indicating how they were calculated

Our web collection on statistics for biologists contains articles on many of the points above.

Software and code

Policy information about availability of computer code

Data collection	No software was used to collect the data. Data was available via direct access to a healthcare plan administrative database.
Data analysis	List of all analysis Python libraries is provided in the environment.yml file in the repo and attached code.

For manuscripts utilizing custom algorithms or software that are central to the research but not yet described in published literature, software must be made available to editors and reviewers. We strongly encourage code deposition in a community repository (e.g. GitHub). See the Nature Portfolio guidelines for submitting code & software for further information.

Data

Policy information about availability of data

All manuscripts must include a data availability statement. This statement should provide the following information, where applicable:

- Accession codes, unique identifiers, or web links for publicly available datasets
- A description of any restrictions on data availability
- For clinical datasets or third party data, please ensure that the statement adheres to our policy

The raw data data are protected and are not available due to data privacy laws and commercial interests. Investigators with an academic affiliation may contact the corresponding author for data access for the purposes of validating the above findings. Requests will be processed within 60 days.

Field-specific reporting

Please select the one below that is the best fit for your research. If you are not sure, read the appropriate sections before making your selection.

☒ Life sciences ☐ Behavioural & social sciences ☐ Ecological, evolutionary & environmental sciences

For a reference copy of the document with all sections, see nature.com/documents/nr-reporting-summary-flat.pdf

Life sciences study design

All studies must disclose on these points even when the disclosure is negative.

Sample size	All patients meeting the inclusion criteria were included. A full list of inclusion criteria can be found in the manuscript.
Data exclusions	Data exclusion criteria were designed by a board certified endocrinologist, to remove any patients with diabetes other than type 2. A full list of exclusion criteria can be found in the manuscript.
Replication	At the start of the study 20% of the patients that met the inclusion criteria were set aside (ATEs and rankings were calculated using the other 80% of the data) and not used for average treatment effect estimates of treatment rankings. The consistency of the rankings was validated using the held-out dataset.
Randomization	Randomization was performed using standard stratified random sampling as implemented in scikit-learn (Python).
Blinding	Only observational (retrospective) data was used. Authors who performed the analysis did not have access to the held-out dataset until the end of the study.

Reporting for specific materials, systems and methods

We require information from authors about some types of materials, experimental systems and methods used in many studies. Here, indicate whether each material, system or method listed is relevant to your study. If you are not sure if a list item applies to your research, read the appropriate section before selecting a response.

Materials & experimental systems

n/a	Involved in the study	
☒	☐	Antibodies
☒	☐	Eukaryotic cell lines
☒	☐	Palaeontology and archaeology
☒	☐	Animals and other organisms
☒	☐	Human research participants
☒	☐	Clinical data
☒	☐	Dual use research of concern

Methods

n/a	Involved in the study	
☒	☐	ChIP-seq
☒	☐	Flow cytometry
☒	☐	MRI-based neuroimaging
